# Computer Vision for Monitoring Wild Bees and Wasps: A Structured Literature Review

**DOI:** 10.1002/ece3.73794

**Published:** 2026-06-30

**Authors:** Chenchang Liu, Patrick Mäder, Marco Seeland

**Affiliations:** ^1^ Department of Computer Science and Automation Technische Universität Ilmenau Ilmenau Germany; ^2^ Faculty of Biological Sciences Friedrich‐Schiller‐Universität Jena Jena Germany; ^3^ German Centre for Integrative Biodiversity Research (iDiv) Leipzig Germany

**Keywords:** computer vision, deep learning, ecological monitoring, nature conservation

## Abstract

Wild bees and wasps are vital to ecosystems, yet large‐scale monitoring of individual insects as well as their habitats and behaviors requires expert knowledge and is very labour‐intensive. Recent advances in computer vision offer both methodological solutions and practical applications for these tasks. This review systematically surveys state‐of‐the‐art literature (2020–2026) on computer‐vision‐based monitoring of wild bees and wasps. We compare the primary monitoring tasks, such as individual detection and classification, habitat observation, and assessment of insect behavior, and analyze how specific computer vision techniques contribute to each. By examining the datasets used in the reviewed studies, we further categorize dataset types and collection strategies to inform future image acquisition. In addition, we draw insights from widely used public resources (e.g., iNaturalist, Observation) regarding their strengths and limitations for this domain. We then examine how hardware and software are integrated in these studies, and review released repositories, existing applications, and the design of more complex, multifunctional monitoring stations. These analyses provide guidance for benchmarking and future deployment with existing datasets and monitoring tools. We eventually propose the design of building more comprehensive and efficient automatic monitoring systems for wild bees and wasps.

## Graphical abstract

This review surveys recent (2020–2026) computer vision research on automated monitoring of wild bees and wasps, covering tasks such as species detection, habitat observation, and behavior analysis. It analyzes datasets, public resources, hardware, and software development. It proposes future directions for building more comprehensive and efficient automated monitoring systems.

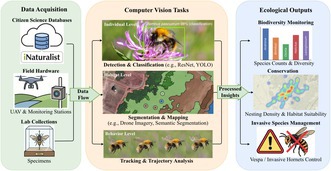

## 1 Introduction

The global decline in insect biomass (Hallmann et al. 2017) and wild bee populations (Zattara and Aizen 2021) has raised serious concerns about biodiversity loss and ecosystem stability (Wagner 2022). Wild insects, including bees and wasps, are essential components of ecosystems, contributing to key functions such as pollination and pest control. Understanding the drivers of their population decline and their responses to environmental change requires reliable and efficient monitoring approaches. Recent advances in deep learning have transformed the field of computer vision, enabling remarkable progress in tasks like classification, detection, and segmentation (Voulodimos et al. 2018). Applying these techniques to ecological monitoring has opened new opportunities for automated monitoring of insects and their habitats (Bjerge et al. 2023; Roy et al. 2024). Deep‐learning‐based computer vision methods have shown strong potential for insect monitoring tasks that are difficult to address using traditional manual approaches, including automated detection, classification, and behavioral observation (Bilik et al. 2024). Current studies on wild bee and wasp monitoring present a large variety of ecological objectives, taxonomic scopes, methodological choices, and dataset designs. This heterogeneity makes it difficult to obtain a coherent view of this field. We found that existing research on computer vision for bee and wasp monitoring remains fragmented in several important ways:

1. The taxonomic scope and ecological framing of many studies are inconsistent. Publications often operate at relatively coarse taxonomic levels or combine bees and wasps with broader insect groups, while the ecological rationale for these choices is not always clearly articulated. As a result, it remains difficult to compare studies in terms of species coverage, ecological objectives, and image context.
2. The dataset landscape has not yet been systematically synthesized. Although studies draw on field observations, citizen‐science platforms, laboratory images, and benchmark datasets, their geographical coverage, taxonomic representation, and practical limitations remain insufficiently compared.
3. A wide range of computer vision methods has been applied, but their respective strengths, limitations, and suitability for different ecological tasks have not been clearly organized. This makes it difficult to determine which approaches are most appropriate for classification, detection, habitat analysis, and behavioral monitoring.
4. Existing applications, repositories, and monitoring platforms remain scattered, and their underlying software and hardware design choices have not been reviewed in an integrated manner.

In addition, the intrinsic taxonomic complexity of wild bees and wasps highlights an important limitation of image‐based monitoring alone, emphasizing the need to combine computer vision approaches with taxonomic expertise and genetic methods such as DNA‐based identification (Høye et al. 2021; Smith et al. 2024).

Despite these limitations, computer vision remains highly relevant for ecological research. A comprehensive review is therefore needed to critically assess recent publications, clarify the ecological and methodological landscape, identify existing gaps, and guide the development of more robust monitoring systems for wild bees and wasps. The main objectives of this review include:

1. Review recent (2020–2026) research on computer‐vision‐based monitoring of wild bees and wasps and summarize the corresponding publication venues and publication types.
2. Categorize the selected studies according to their taxonomic scope, ecological objectives, and image data context.
3. Analyze dataset characteristics, including geographical coverage, taxonomic representation, image context, and the availability of public benchmark resources.
4. Compare the computer vision methods used across studies and assess their suitability for different ecological tasks, including classification, detection, habitat analysis, and behavioral monitoring.
5. Examine existing repositories, applications, and monitoring platforms, with particular attention to their underlying software and hardware designs.
6. Derive structured guidance for researchers developing more robust and comprehensive monitoring systems for wild bees and wasps.

## 2 Literature Search

The literature search followed several main steps. An initial set of seed papers was selected as the starting point, and the literature set was then expanded through both forward and backward snowballing based on these papers. The retrieved studies were manually reviewed, and the most relevant publications were retained according to predefined criteria to finalize the selection list. Key information was then extracted from the selected studies in line with the main objectives of this review and the proposed research questions.

The seed papers are identified through a manual search on *Google Scholar* using relevant keywords, followed by the manual selection of recent, highly cited, and influential publications. The keywords are shown in Table 1. Given that this review addresses a relatively specific and still emerging research area, we initially selected 18 seed papers to improve coverage. Among them, 3 publications with relatively high citation counts (≥ 20) were chosen as the initial papers for the snowballing process. These 3 papers were required to be published in leading journals or conference proceedings and to be indexed in at least one of the following scholarly databases: *Web of Science*, *IEEE Xplore*, *ScienceDirect (Elsevier)*, or the *ACM Digital Library*. The remaining seed papers were retained in the candidate pool and carried forward to the subsequent stages of duplicate removal and eligibility screening.

**TABLE 1:** Keywords to identify seed papers.

Focus	Keywords
Taxa	Wild, bee, wasp, Hymenoptera, wing
Habitat	Habitat, environment, hive, nest
Behavior	Aggregation, activity, health, parasite
Methodology	Deep learning, neural network, computer vision, detection, classification, tracking, monitoring, segmentation, identification, recognition

Backward snowballing begins with a seed paper and examines its reference list to identify earlier studies that are relevant. Any qualifying referenced paper is added to the seed paper set, and the process is iteratively repeated on each newly added publication. In contrast, forward snowballing starts with a seed paper and identifies subsequent publications that cite it. Relevant citing papers are incorporated into the seed set, and the process is similarly iterated.

**FIGURE 1:**
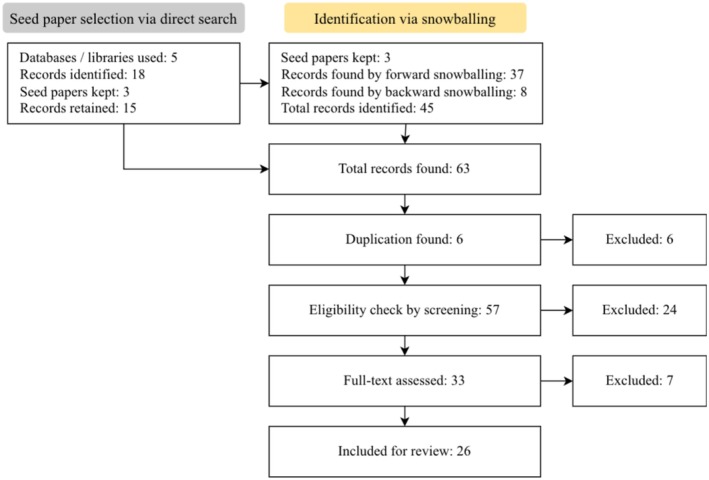
Workflow of the literature identification and selection process, including direct database search for seed papers and subsequent snowballing.

After removing duplicate records, the remaining publications were screened based on title and abstract, followed by full‐text assessment for eligibility. Only publications that matched the scope of this review were retained. The overall workflow of the selection process is illustrated in Figure 1. In total, more than 60 studies were initially identified, but only 26 were ultimately included after applying our selection criteria: We retained only papers that focused on wild bees rather than domesticated bees and that employed computer vision methods using images or videos as input. Publications based on datasets containing only a small proportion of bee images, while primarily addressing other insect taxa, were excluded. We also limited the review to recent studies published between 2020 and 2026. Survey papers were excluded because they did not introduce new methodologies, and unpublished or non‐peer‐reviewed articles were not considered. In addition, a small number of papers were excluded because of unclear dataset descriptions or the use of non‐natural images. The main reason for exclusion is that many studies rely on datasets focusing on images of domesticated bees and managed hives, whereas our focus is on wild bees and wasps. In cases where datasets include both domesticated and wild bees, we retained only those containing at least one wild bee or wasp taxon at the *Genus* or *Species* level.

### 2.1 Research Questions

Given the objectives outlined in Section 1, we formulate the following research questions to guide our systematic review:

- **RQ1 (Categorization):** How can the selected publications be categorized in terms of species coverage, ecological objective, and image data context?
- **RQ2 (Datasets):** What are the geographical distributions of wild bee and wasp species represented in the datasets, and which benchmark datasets are publicly available?
- **RQ3 (Methods):** What computer vision approaches are employed, and what ecological tasks do they solve?
- **RQ4 (Applications and Tools):** What monitoring tools, platforms, or applications have been developed, and what are their underlying design principles?

## 3 Results

**FIGURE 2:**
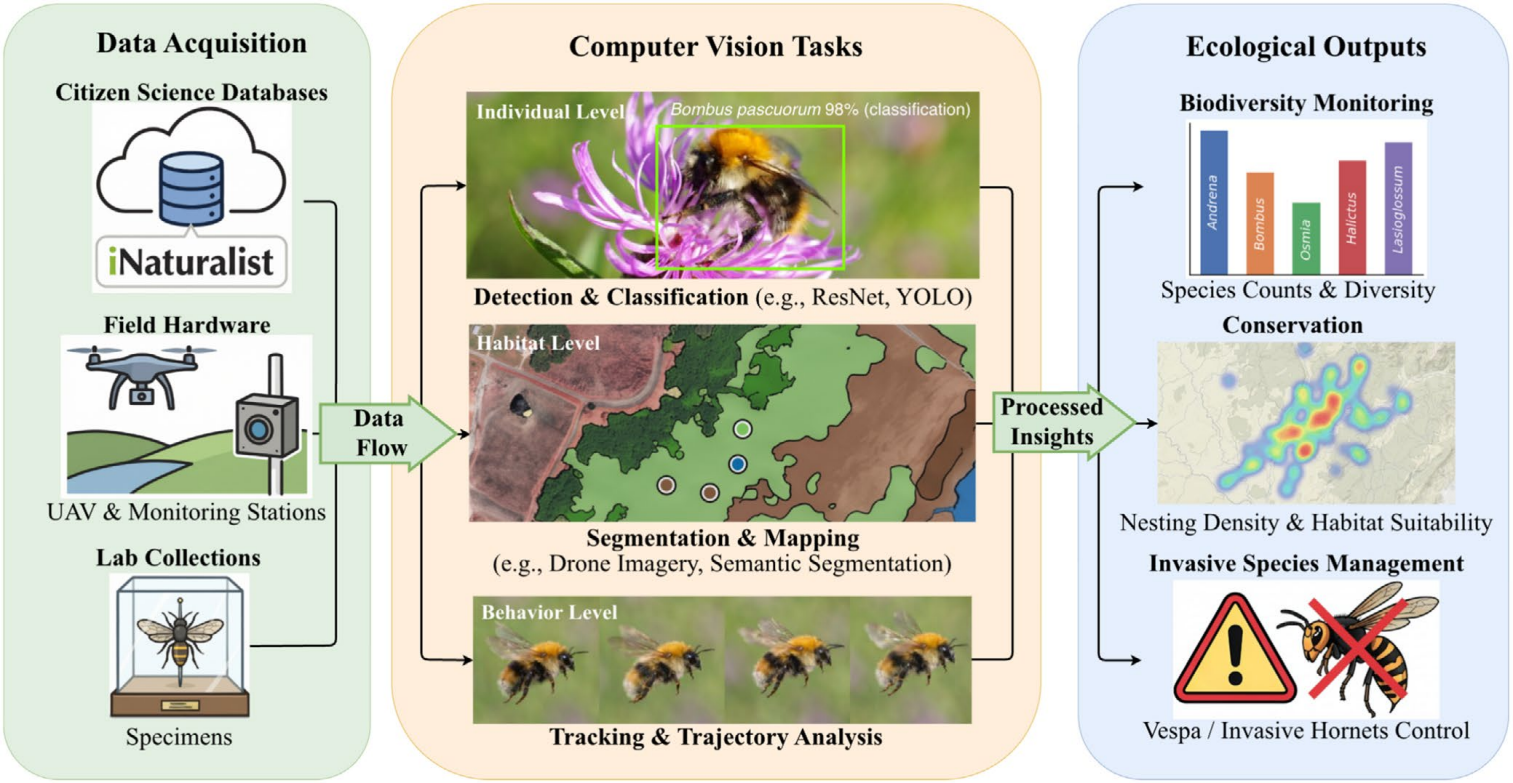
The ecological pipeline of computer vision‐based monitoring for wild bees and wasps. The workflow aggregates the three primary data sources identified in the review (panel left), including citizen science platforms and field hardware. These inputs are processed through distinct computer vision tasks at the individual, habitat, and behavioral levels (panel middle), which are finally mapped to their ultimate ecological objectives (panel right), such as biodiversity monitoring and invasive species management.

In this section, we present the results of our review based on the 26 selected papers. We systematically analyze the temporal and geographic distribution of publications, publication venue types, dataset characteristics, taxonomic focus, monitoring tasks, computer vision methods, and existing applications and tools. To provide a structured overview of the reviewed literature, Figure 2 illustrates the complete monitoring workflow, mapping the progression from initial data acquisition sources to the specific computer vision tasks employed, and finally to their intended ecological applications.

### 3.1 Geographic and Temporal Trends

**FIGURE 3:**
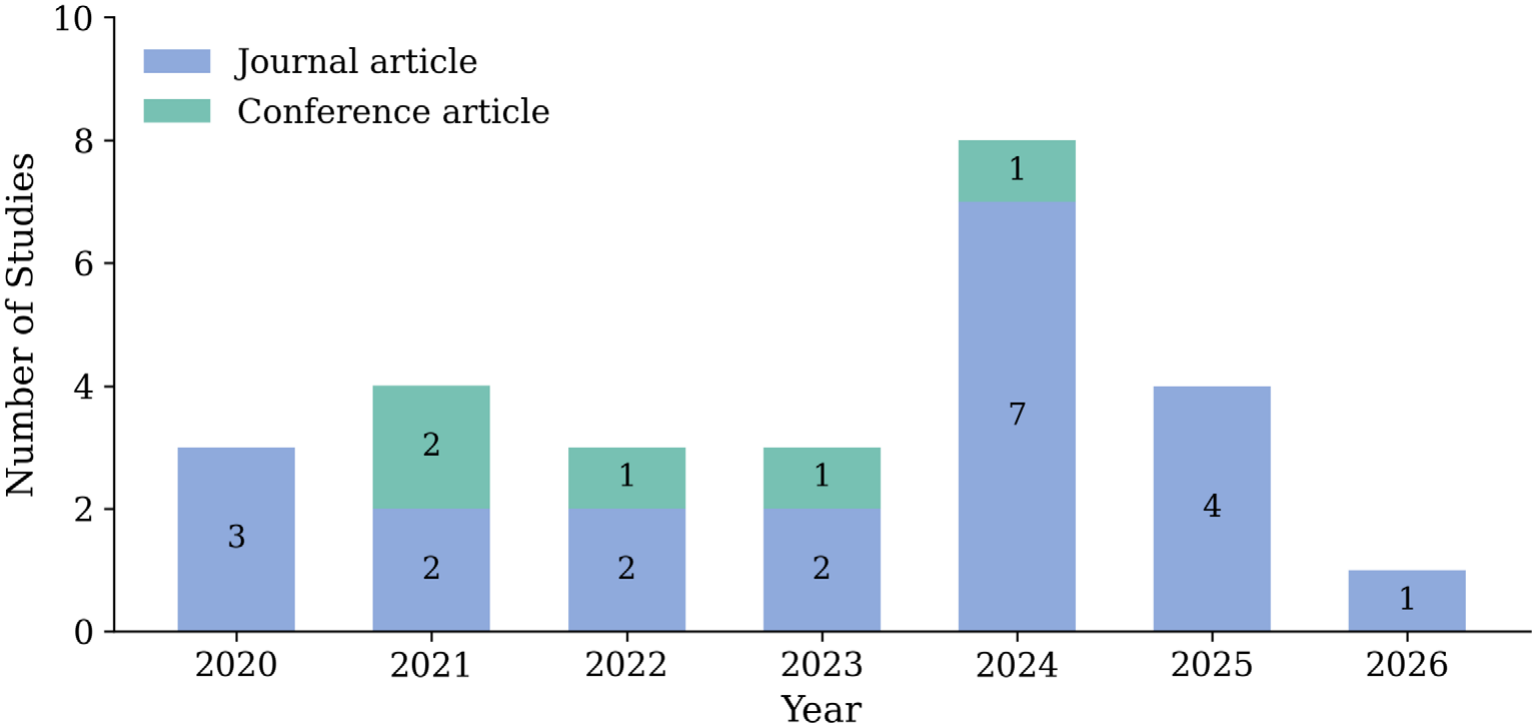
Distribution of the year of publication of the reviewed publications, grouped into journal and conference articles.

To gain an overview of active research groups and their geographical distribution, we analyzed the first author's affiliation across the 26 selected papers. The results show that the majority of the publications originate from Europe (10/26), followed by North America (6/26) and Asia (5/26), Oceania (3/26), and South America (2/26). The United States is the most represented country, contributing 6 papers, followed by Germany (3), Korea (3), and Australia (3). One paper each originates from Spain, Belgium, Italy, Portugal, Switzerland, Hungary, the United Kingdom, China, the Philippines, India, Argentina, and Brazil. The annual distribution of publications from 2020 to 2026 is illustrated in Figure 3, indicating a positive trend in research activity within this domain over the past 5 years.

### 3.2 Dataset Origin and Geographical Diversity

**FIGURE 4:**
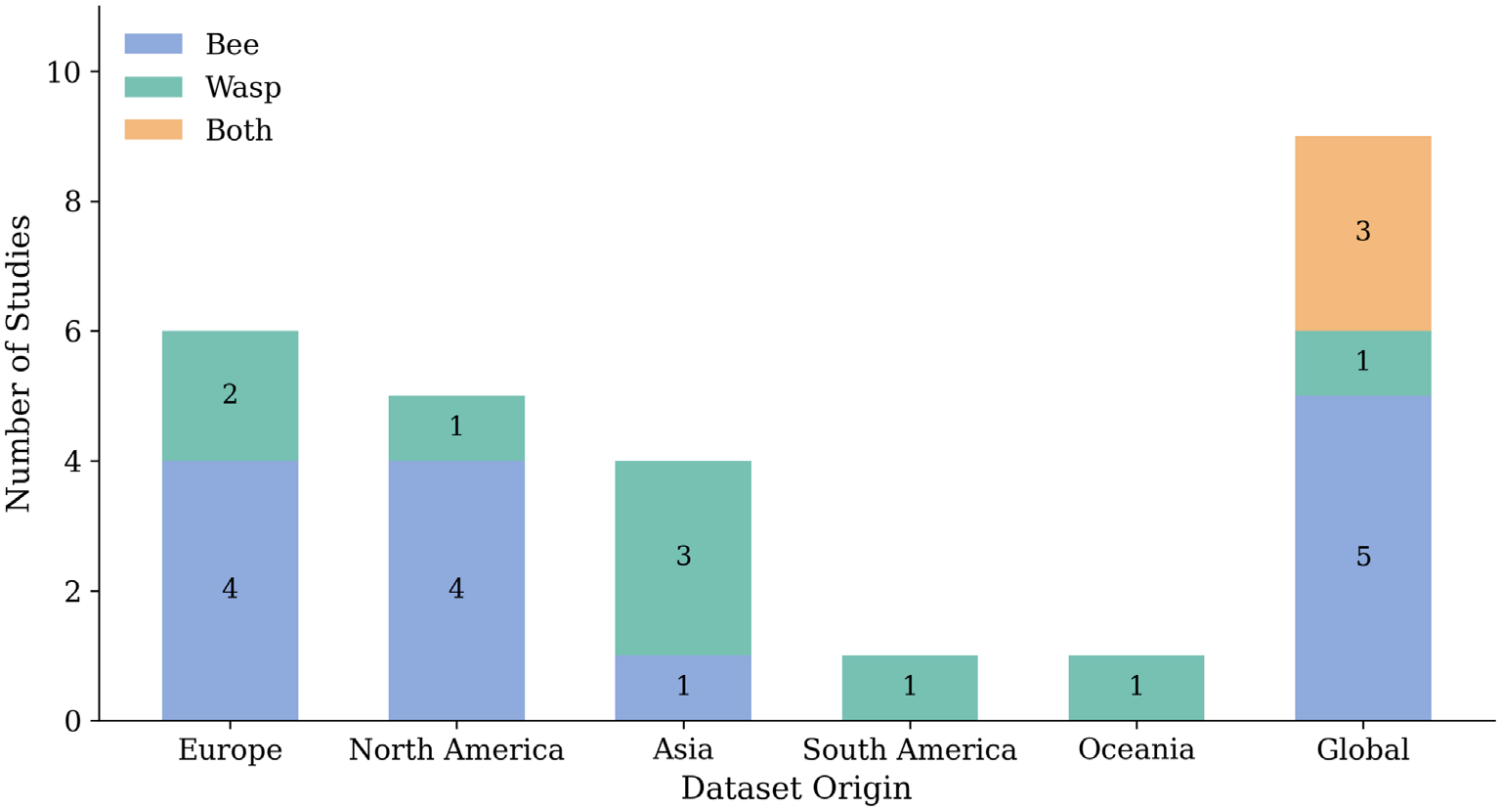
Geographic origins of the datasets used in the reviewed publications. The datasets originated from five continents: Europe, North America, Asia, South America, and Oceania. Datasets for which no specific location was reported, or that were obtained from global citizen science platforms, are marked as “Global”.

To assess dataset origins and geographical diversity, we examined the data collection locations reported in the selected papers, as illustrated in Figure 4. The results show that most datasets were collected in Europe (6/26), followed by North America (5/26), Asia (4/26), South America (1/26), and Oceania (1/26). The United States is the most frequent data source, contributing to 5 studies, followed by Korea (3), Germany (2), and Portugal (2). The countries that appeared only once are the Netherlands, Switzerland, UK, Jersey, France, Canada, India, Argentina, and Australia. In 9 studies, data were obtained from global citizen science platforms like iNaturalist (n.d.), where collection sites were either diverse across multiple countries or not explicitly specified.

Among all the datasets, 16 contain images of individual wild bees or wasps, while 3 datasets specifically focus on bee wings. Seven datasets include images of habitats: Four with images of nests or hives, one focuses on flowers, and another on trees that are frequently visited by wild bees. In addition, one dataset focuses on the surrounding environments of wild bees, with both natural and anthropogenic backgrounds where the images were captured.

### 3.3 Taxonomic Focus

**FIGURE 5:**
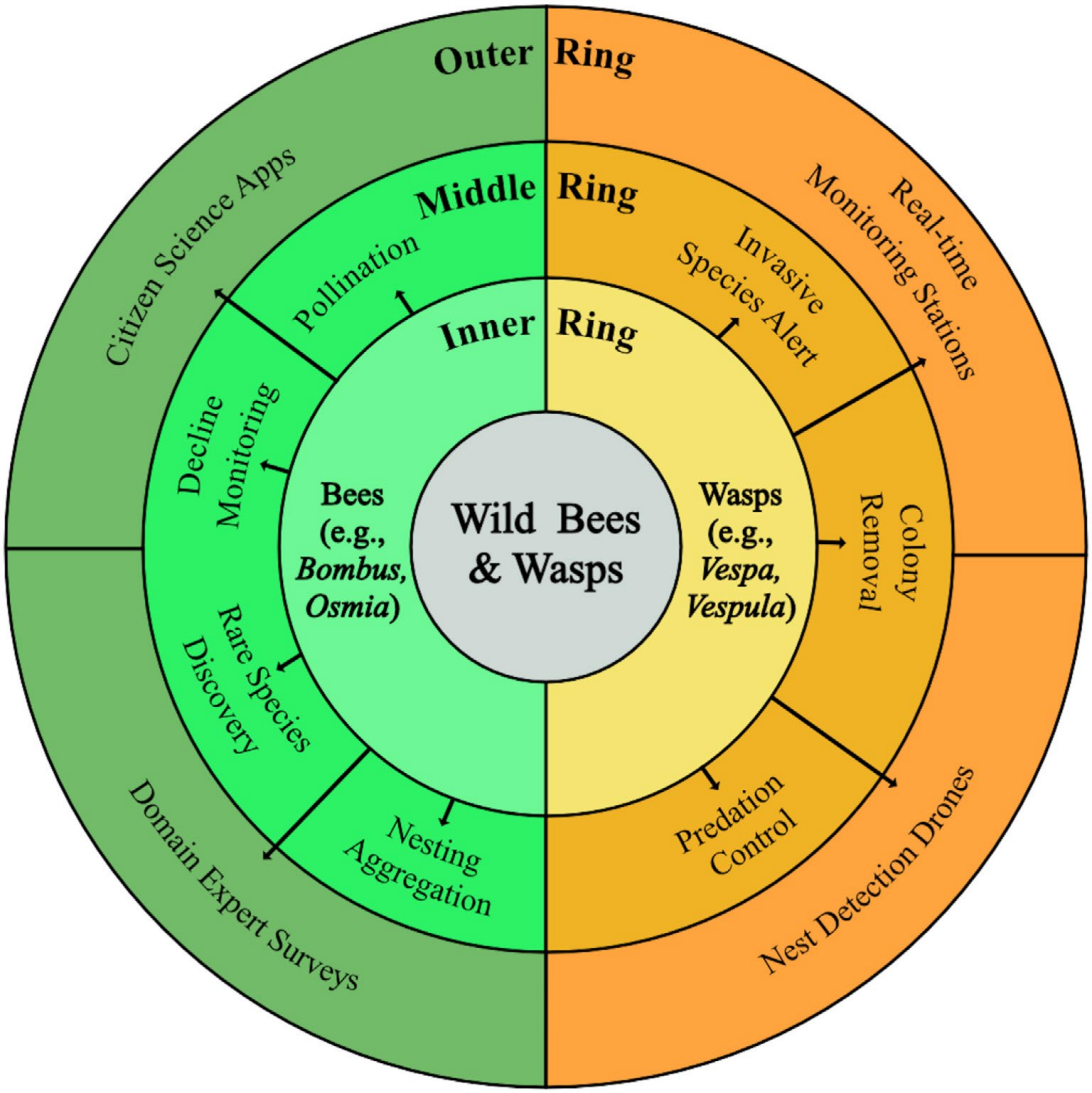
Taxonomic focus and associated ecological objectives. The diagram illustrates the divergence in monitoring goals: Wild bee research (left) is primarily driven by conservation needs such as pollination monitoring and addressing population decline, while wasp research (right) heavily emphasizes pest control and invasive species management, particularly for the genus *Vespa*. The inner ring depicts the taxonomic focus, the middle ring the ecological objectives, and the outer ring the technologies employed.

Out of 26 studies, 14 exclusively focus on wild bees, 9 on wasps, and 3 on both wild bees and wasps. The distinct ecological motivations driving research for different taxa are visualized in Figure 5, which contrasts the conservation‐focused objectives for wild bees against the control‐focused objectives for wasps.

#### 3.3.1 Wild Bees

Roughly half of the reviewed studies focus on wild bees (14/26). All of these publications highlight the ongoing global decline of wild bee populations. As wild bees are the primary pollinators of many ecosystems and agricultural crops, their alarming decline has become a major ecological concern. A key step in investigating this issue is the accurate identification of wild bee taxa. Several studies (Bhuiyan et al. 2022; Buschbacher et al. 2020; Buschbacher and Steinhage 2020; Hossain et al. 2021; Kelley et al. 2021; Knauer et al. 2022b; Spiesman et al. 2024, 2021; Srinivas et al. 2025; Varga‐Szilay et al. 2024; Yoo et al. 2023) have addressed classification or detection at the genus or species level. Among them, some studies (Bhuiyan et al. 2022; Yoo et al. 2023) also aim to recognize non‐bee insects or bee mimics, and to detect health indicators such as *Varroa* mites or the presence of pollen. In addition, wing morphological features such as veins, cells, and junctions are analyzed in publications such as (Buschbacher and Steinhage 2020) to evaluate the contribution of these traits to species identification.

Wild bee habitat studies often focus on nesting behavior and aggregation patterns. Wild bees rely on nectar and pollen from flowers as their primary food sources, and the majority of the species are solitary, ground‐nesting bees (Kueneman et al. 2024; Orr et al. 2022). Therefore, floral cover, nesting density, and tree distributions could be important indicators of bee abundance (Grundel et al. 2010). Computer vision methods support analysis of these indicators, thereby contributing to broader ecological investigations, such as of habitat use, resource availability, and population distribution (Chieffallo et al. 2025; Potts et al. 2003). As their life cycles depend heavily on these floral resources, several studies (Chieffallo et al. 2025; Mueller and Buckner 2025; Neyns et al. 2025) have employed drone imagery to investigate the relationships between wild bee nesting sites and the distribution of flowers or trees from above.

#### 3.3.2 Wasps

Nine of the reviewed studies (9/26) focus on wasps. As members of the order *Hymenoptera*, wasps provide important ecosystem services. These include regulating arthropod populations, pollination, supporting processes such as wasp‐mediated seed dispersal, and serving as bioindicators of environmental conditions (Borchardt et al. 2024; Brock et al. 2021). Despite these ecological roles, the reviewed publications mainly investigate them as natural enemies of domesticated bees such as *Apis mellifera* . In this computer‐vision‐based research, species such as *Vespa mandarinia* and *Vespa velutina* are framed as invasive or harmful because of their predation on bee colonies, their potential risk to humans, and their disruptive impacts on local ecosystems (Guangnan and Zhenyou 2022; Jeon et al. 2023). Several studies (Guangnan and Zhenyou 2022; Jeon et al. 2023; Kwon et al. 2024; María‐Luisa and Jesús‐Ángel 2024) have developed real‐time wasp monitoring and alert systems to support honeybee health, pest management, and wasp removal planning. Some wasps demonstrate parasitic behavior, such as the *Diapriidae* family. Shirali et al. (2024) developed methods for detecting these parasitoid wasps in lab‐quality images.

Habitat‐level studies contribute to the control of wasp nesting locations and behaviors. Singha Roy et al. (2023) analyzed wasp microhabitats through image background classification and concluded that many wasp species adapt well to urban environments. Kim et al. (2025) investigated potential wasp hive site detection using UAV‐captured images, while Martínez et al. (2024) and O'Shea‐Wheller et al. (2024) monitored nest traffic and activity patterns using autonomous monitoring stations for early detection and alerting. These efforts collectively contribute to preventing agricultural damage and improving pest control strategies.

Wasps frequently co‐occur with insects such as bees, and they are often misidentified as other insect species. The study from Braga and Madureira (2020) applied computer vision methods to differentiate *Vespa velutina* from co‐occurring bees, including *Apis mellifera* and *Bombus terrestris* . Chiranjeevi et al. (2024) developed InsectNet, a large‐scale insect species classification model trained with self‐supervised learning (SSL) on citizen‐science images covering 2526 insect species, including a large number of wild bees and wasps. Rebelo et al. (2021) introduced a pipeline that segments wing images of both bees and wasps and extracts skeleton and vein‐junction landmarks for species classification.

#### 3.3.3 Frequently Studied Genera

Eleven publications (12/26) focus on a single bee or wasp genus, although each genus may include data from multiple species. Among these, four publications study bee genera, namely *Bombus* (3/26), *Osmia* (1/26), and *Andrena* (1/26). Seven publications focus on wasp genera, with *Vespa* represented in 6 studies and *Vespula* in 1 study. The remaining publications cover multiple genera or broader taxonomic levels.

Bumblebees (*Bombus*) play a crucial role in pollinating both wildflowers and agricultural crops (Kleijn et al. 2015; Rao and Stephen 2009; Varga‐Szilay et al. 2024). At the same time, they are strongly affected by the global decline of wild bee populations (Zattara and Aizen 2021). Bhuiyan et al. (2022) focused on distinguishing *Bombus* from non‐*Bombus* species, classifying bees and bumblebees to evaluate how effectively various bee mimics can deceive AI models. Varga‐Szilay et al. (2024) studied the foraging behavior of *Bombus terrestris* on flowers. Spiesman et al. (2021) compiled a large bumblebee image dataset from citizen science sources and experimented on the identification of 36 diverse *Bombus* species.

*Osmia* is a genus of solitary bees commonly known as mason bees. Species of *Osmia* exhibit a wide range of nesting habits, often occupying preexisting cavities or old nests created by other insects, crevices or holes beneath or between grass tussocks or stones, spaces beneath bark, and hollow stems (Rightmyer et al. 2013). Using *Osmia* as an example, Knauer et al. (2022a) proposed an open‐source pipeline for the behavioral monitoring of solitary bees. The pipeline includes functions such as detecting bees and nest cavities and reading individual ID tags.

*Andrena vaga* is a ground‐nesting mining bee. Despite being a solitary species, *Andrena vaga* often nests in large aggregations, where hundreds to thousands of individual nests are locally concentrated (Neyns et al. 2025; Westrich 2019). Neyns et al. (2025) investigated the spatial relationship between the nesting sites of *Andrena vaga* and the distribution of *Salix* trees, which provide an essential pollen and nectar source for this and other bee species.

*Vespa* is the wasp genus receiving particular attention, as it is typically recognized as a major predator of domesticated bees. Existing studies on *Vespa* mainly focus on rapid detection and nest removal strategies. Kwon et al. (2024) introduced an improved YOLOX model to enhance accuracy and speed in small‐object detection of *Vespa* hornets. Jeon et al. (2023) similarly developed a portable, real‐time hornet detection and alert system for *Vespa* based on the YOLOv5 architecture. O'Shea‐Wheller et al. (2024) designed a hardware‐assisted AI pipeline combining YOLOv5s and ResNet‐50 to detect *Vespa* at bait stations in real time. Guangnan and Zhenyou (2022) proposed a framework for predicting the nesting locations of *Vespa mandarinia*. María‐Luisa and Jesús‐Ángel (2024) built and compared four CNN (convolutional neural networks, see Section 3.4.1) classifiers for the identification of *Vespa velutina*. Finally, Braga and Madureira (2020) employed Mask R‐CNN to detect *Vespa velutina*, supporting rapid response and nest removal planning.

Another wasp genus represented in the reviewed publications is *Vespula*. Some species, such as *Vespula germanica*, threaten agriculture and silviculture and can negatively affect biodiversity because of their polyphagous habits. In addition, their aggressive behavior and venomous stings can pose risks to human health (de Villiers et al. 2017). Some *Vespula* species are considered invasive outside their native range. For example, *Vespula germanica* and *Vespula vulgaris* are native to Europe, although both species have become widespread and abundant in several continents worldwide (Lester and Beggs 2019). Martínez et al. (2024) studied colonies of *Vespula* and developed an autonomous monitoring station that records nest traffic to classify castes such as worker, drone, or gyne, and movement direction. Their system also enables behavioral monitoring of *Vespula* colonies alongside environmental covariates such as temperature and humidity, contributing to effective control of harmful social insect populations.

### 3.4 Monitoring Tasks

Considerable research in deep‐learning‐based computer vision has led to significant advances in automated insect monitoring and a growing number of publications in recent years (Wäldchen and Mäder 2018). Deep learning‐based computer vision methods involve the design of neural networks with multiple layers to foster the ability to extract features from raw data. These models perform tasks such as image classification, object detection, and segmentation by recognizing visual patterns and discovering underlying feature representations within input images. Such capabilities make them well suited for monitoring wild insects like bees and wasps and their habitats, where visual characteristics such as shape, color, and movement would otherwise require significant human effort to analyze (Christin et al. 2019).

**FIGURE 6:**
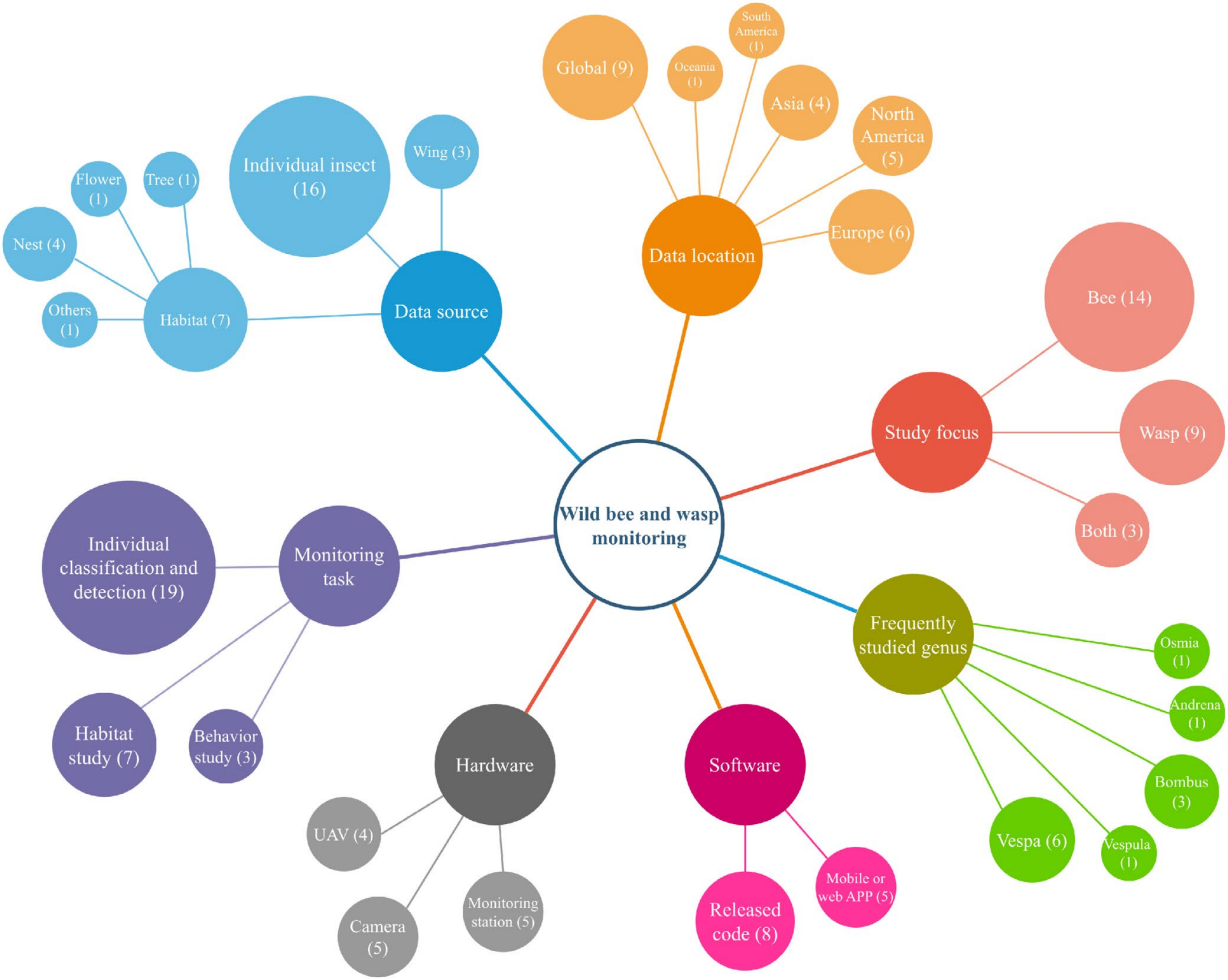
Mind map of the selected publications. Nodes in different colors represent different types of findings reported in the reviewed publications. The sizes of the terminal nodes correspond to the number of publications addressing each focus area, and the numbers inside indicate the exact counts.

Choosing a suitable deep learning model for ecological tasks should take into account factors such as speed, accuracy, computational resources, and the deployment environment (Nawoya et al. 2024). In this section, we conduct a systematic analysis of how different deep learning methods are applied across diverse task categories. After examining the selected publications, we categorize the research focus into several key tasks and analyze how computer vision methods are applied in each, as shown in the purple nodes in Figure 6: individual classification and detection (19 publications), habitat study (7 publications), behavior study (3 publications). The node sizes in the mind map correspond to the number of studies addressing each focus area. There are overlaps among these study focuses, meaning that some publications address multiple tasks simultaneously.

#### 3.4.1 Classification and Detection of Individual Insects

**FIGURE 7:**
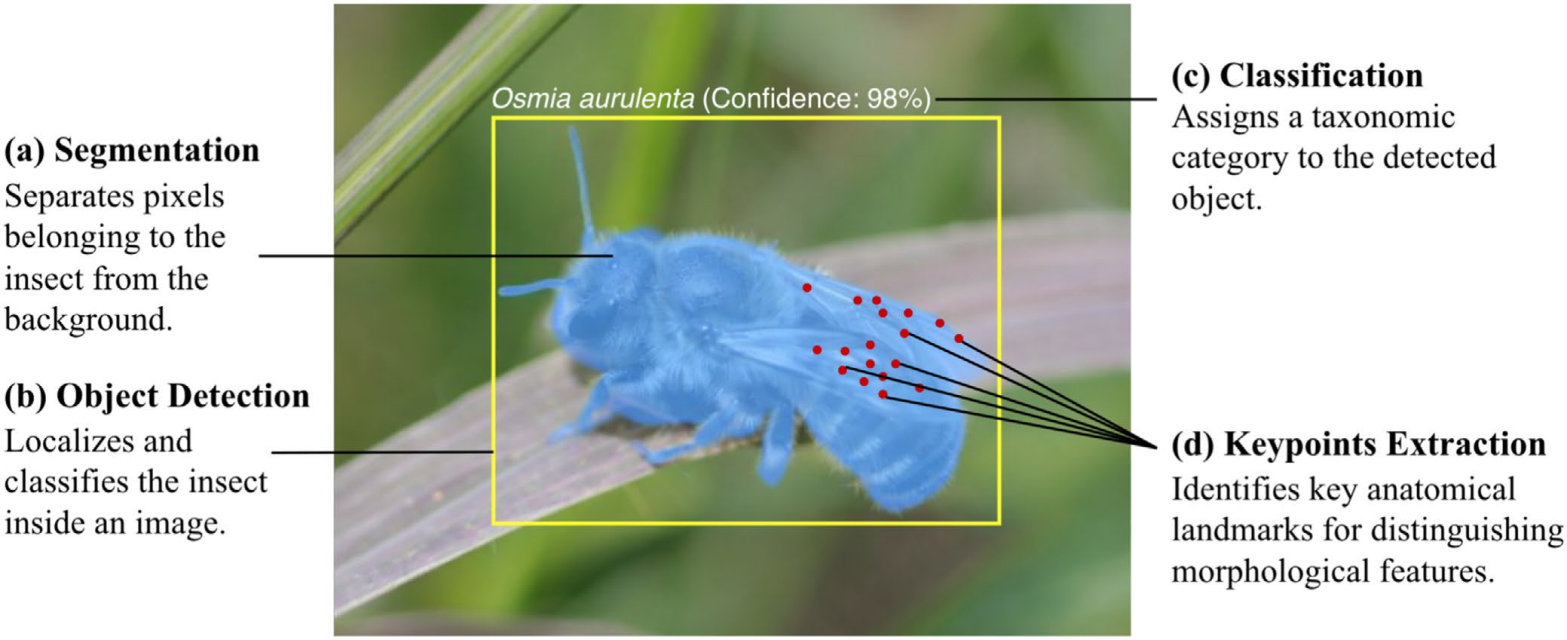
Visualizing the primary computer vision tasks in ecological monitoring. The annotation illustrates (a) Instance Segmentation (blue mask) for precise morphological analysis, (b) Object Detection (yellow bounding box) for localization, (c) Classification (label) for taxonomic identification, which is the most frequent task, and (d) Keypoint Extraction (red dots) for identifying specific traits like wing vein junctions.

We categorize the primary computer vision methodologies applied in these studies into four distinct tasks, which are visually defined in Figure 7 using a representative wild bee image.

Among the 26 reviewed studies, the majority (19/26) focus on the level of individual insects, such as detecting and classifying bee or wasp instances in images.

Eighteen publications belonging to this type of task (18/26) incorporated the use of convolutional neural networks (CNNs). CNNs are widely used in computer vision research and applications due to their effectiveness in visual‐data processing (Ratnayake et al. 2024; Yıldız and Karabağ 2025). A CNN consists of convolutional filters (also called “kernels”) with learnable weights and biases (LeCun et al. 2010). The convolutional layers in a CNN apply these filters to the input data to produce feature maps that capture spatial hierarchies and patterns.

Commonly used CNN‐based models in these 18 publications include ResNet, YOLO, R‐CNN, and MobileNet. Other CNN models, such as VGG, EfficientNet, and Inception, also appeared, but each was used in fewer than three publications. Detailed information on the models used in each study is provided in the Supporting Information. One publication (1/26) combines CNN and vision transformer (ViT) (Dosovitskiy et al. 2020). Transformer architectures, originally developed for natural language processing tasks, have rapidly gained prominence in computer vision. ViT models introduce a novel paradigm by operating on sequences of image patches, which enables interactions among different regions of an image and achieves strong performance on classification tasks (Dosovitskiy et al. 2020; Pereira and Hussain 2024). When pre‐trained on large datasets and transferred to mid‐sized or smaller benchmarks, ViT models achieve accuracy comparable to state‐of‐the‐art convolutional networks while requiring substantially fewer computational resources for training (Dosovitskiy et al. 2020).

One other publication (1/26) from this task category uses a classical computer vision and machine learning pipeline rather than a deep‐learning approach. However, it is a relatively recent study (2021) and provides important ecological insights that align well with the focus of our review. For example, it focuses on bee wing images and examines morphological characteristics such as vein junctions. The authors also note that their approach has the potential to be improved using more sophisticated landmark classifiers in future work (Rebelo et al. 2021).

##### 3.4.1.1 Residual Neural Network (ResNet)

ResNet is the most frequently used architecture, appearing in 9 studies for individual‐level classification and detection tasks. He et al. (2015) proposed ResNet in 2015. ResNet is a deep CNN architecture that introduces residual learning to address the difficulties of training very deep models, that is, models with many layers. Its key innovation is the residual block, which uses skip connections. This design allows the network to learn residual functions instead of identity mappings. Residual learning eases the optimization of deeper networks and enables them to achieve higher accuracy as depth increases.

ResNet50 was used as a feature extractor by Yoo et al. (2023) to classify bee species, reject non‐bee images, and detect health conditions depending on the presence of pollen or varroa. Srinivas et al. (2025) included ResNet50 as one of the benchmark models for species classification, while O'Shea‐Wheller et al. (2024) employed ResNet50 for detecting *Vespa velutina* and *Vespa crabro* at bait stations.

Kelley et al. (2021) proposed a pipeline for analyzing uncropped crowdsourced photographs. This pipeline detects two bee genera and their associated species, including both wild and domesticated bees, using a ResNet101 backbone with a feature pyramid network (FPN) and classifies three species commonly found in the Midwestern United States. Although the study includes the domesticated bee *Apis mellifera* , it also explicitly considers two wild bee species, *Bombus griseocollis* and *Bombus impatiens* . We therefore retained it among the reviewed publications. Bhuiyan et al. (2022) similarly used ResNet101, trained on iNaturalist images, to classify bee species from eight genera and to evaluate how well bee and wasp mimics across 7 insect families (Asilidae, Bombyliidae, Scarabaeidae, Sesiidae, Sphingidae, Syrphidae, Tachinidae) deceive the automated classifiers. Spiesman et al. (2021) used both ResNet101 and Wide‐ResNet101 for bumblebee species identification and analyzed species‐wise error patterns in relation to sample size.

Chiranjeevi et al. (2024) developed InsectNet, a large‐scale insect species classifier covering 2526 taxa, trained using a self‐supervised learning approach on citizen science images. Their framework used both ResNet50 and RegNetY‐32 for self‐supervised pretraining. Braga and Madureira (2020) evaluated both ResNet101 and ResNet50 as backbones for automated detection and alerting of *Vespa velutina*.

Hossain et al. (2021) compared several models from the ResNet family, including ResNet101e, TResNet, CSPResNet50, and ResNetv2, using a dataset of eleven bee species sampled from iNaturalist. Their study examined which morphological regions drive predictions and compared these to visual features from traditional identification keys used by human experts.

##### 3.4.1.2 You Only Look Once (YOLO)

In 7 studies, YOLO was used to detect individual insects. YOLO was first introduced by Redmon et al. (2016) as a fully convolutional, one‐stage object detector. The model applies a single neural network to the entire input image and divides the image into a grid of cells, with each cell predicting bounding boxes and associated class scores. By performing localization and classification jointly within a single forward pass, YOLO achieves real‐time object detection while maintaining competitive accuracy (Zou et al. 2023). Since its initial release, several improved versions of YOLO have been developed.

Kwon et al. (2024) implemented YOLOX (Ge et al. 2021), a model that enhances accuracy and speed for small insect detection, with a particular focus on *Vespa* hornets. This real‐time system was developed in the context of monitoring several invasive species belonging to the *Vespa* genus in Korea, where their spread raised serious concerns about managed honey‐bee colonies.

YOLOv8 (Jocher et al. 2023) was used by Shirali et al. (2024) for the recognition of *Diapriidae* parasitoid wasps. These tiny insects (1.2–4.5 mm) were photographed under laboratory conditions and identified using DNA barcoding to provide reliable ground truth for training neural networks. Their approach achieved high genus‐level and sex‐level classification accuracy. Spiesman et al. (2024) employed YOLOv8 for pre‐cropping pinned bee specimens to support species‐level identification analyses. Martínez et al. (2024) recorded nest traffic in *Vespula germanica* colonies and used YOLOv8 to classify caste types, including workers, drones, and gynes.

Knauer et al. (2022b) developed an open‐source pipeline that integrates multiple models to detect bees and nest cavities and to read individual ID tags. Marker tags, consisting of unique color‐digit combinations attached to each bee's thorax, are identified using a YOLOv3 network on each detected bee.

O'Shea‐Wheller et al. (2024) used YOLOv5s to detect *Vespa velutina* and *V. crabro* at bait stations in real time, demonstrating a low‐cost and deployable early‐warning system for invasive hornets. Using the same YOLOv5s model, Jeon et al. (2023) designed a portable, real‐time hornet detection and alert system for apiaries targeting *Vespa velutina*.

##### 3.4.1.3 Region‐Based Convolutional Neural Networks (R‐CNN)

Another popular family of CNN‐based detection models is R‐CNN (Region‐based Convolutional Neural Networks), which appeared in 3 reviewed publications. The original idea was introduced by Girshick et al. (2013). It combines region proposals for localization, followed by feature extraction for object classification and bounding box regression. R‐CNN employs selective search to generate these region proposals, which are candidate image regions likely to contain objects. Each proposed region is then reshaped to a fixed size and passed through another CNN to extract features. These features are subsequently used to classify objects and refine their localization via support vector machines (SVMs) and bounding box regressors (Girshick et al. 2013; Hmidani and Ismaili Alaoui 2022). Later on, Girshick (2015) proposed the Fast R‐CNN model in 2015. Fast R‐CNN applies the convolutional network only once per image to generate feature maps, from which all regions of interest are extracted, rather than running a CNN multiple times for each region (Hmidani and Ismaili Alaoui 2022). Subsequently, Ren et al. (2015) introduced Faster R‐CNN, a modified and more efficient version of Fast R‐CNN. In Faster R‐CNN, a Region Proposal Network (RPN) is integrated into the detection network, sharing convolutional features for both region suggestion and classification. This model takes image feature maps as input and produces a set of object proposals, each associated with an objectness score (Hmidani and Ismaili Alaoui 2022; Ren et al. 2015).

Kelley et al. (2021) employed a standard Faster R‐CNN architecture with features extracted from a ResNet101 backbone. Their method is designed to automatically detect and classify bee species from raw, unprocessed images, and it demonstrates strong effectiveness on crowdsourced, low‐quality data (He et al. 2017). Similarly, Knauer et al. (2022b) applied Faster R‐CNN for bee detection, focusing on the species *Osmia bicornis* .

Braga and Madureira (2020) adopted Mask R‐CNN to detect the wasp species *Vespa velutina*. Mask R‐CNN extends Faster R‐CNN by adding a mask prediction branch that outputs a binary mask for each detected object, enabling pixel‐level instance segmentation (He et al. 2017).

##### 3.4.1.4 MobileNet

MobileNet was used in 3 publications. Howard et al. (2017) proposed the first MobileNet architecture in 2017. MobileNet uses depthwise separable convolutions to construct lightweight deep neural networks. MobileNetV2 (Sandler et al. 2018) follows the same principle of depthwise separable convolutions but introduces two important architectural innovations. The first is the use of linear bottlenecks, which reduces information loss introduced by nonlinear activations within convolutional blocks. The second is the inverted residual structure, which preserves feature information by expanding the intermediate representation before projection (Sandler et al. 2018). MobileNets offer flexibility in selecting compact network configurations that are well‐suited for applications with strict resource constraints, including latency and model size (Dong et al. 2020).

Buschbacher et al. (2020) developed a MobileNetV2‐based end‐to‐end system for image‐based wild bee species identification based on images of their wings. They later extended this work by introducing an explanation module based on Visual Backpropagation (Bojarski et al. 2016), which computes pixel‐wise relevance maps highlighting wing regions associated with morphological traits such as veins, cells, and junctions in relation to species identification (Buschbacher and Steinhage 2020).

Srinivas et al. (2025) constructed a field‐collected South Asian bee image dataset captured under uncontrolled outdoor conditions. Multiple CNN architectures were benchmarked for species classification, with MobileNetV2 achieving the highest accuracy of 98.4%.

##### 3.4.1.5 Other CNN Architectures

Some other CNN architectures also appear in the reviewed publications, each appearing fewer than 3 times. Among others, the most relevant models are VGG, Inception, and EfficientNet. VGG (Simonyan and Zisserman 2014) is a convolutional neural network architecture with a simple and uniform design, using stacks of small convolutional layers to increase depth, and its straightforward structure has made it easy to adopt for many computer vision tasks. Inception (Szegedy et al. 2014) introduces the “Inception module”, which processes multiple filter sizes in parallel to capture information at different spatial scales, enabling the network to learn more complex features. EfficientNet (Tan and Le 2019) is a family of models that scale depth, width, and resolution in a balanced way using a compound coefficient. It achieves state‐of‐the‐art accuracy while remaining computationally efficient across a range of model sizes.

Bhuiyan et al. (2022) used a VGG16‐based CNN to distinguish bees from visually similar bee mimics across 19 species from 6 families. Srinivas et al. (2025) also evaluated VGG16, comparing its performance with other CNN models for distinguishing South Asian bee species and non‐bee insects.

In the publication of Srinivas et al. (2025), InceptionV3 was also tested on South Asian bee species, achieving one of the highest accuracies, exceeding 97%. Spiesman et al. (2021) used InceptionV3 for bumblebee identification and concluded that it offered the best speed–accuracy trade‐off for classifying 36 species.

Spiesman et al. (2024) applied EfficientNet for species classification of small and visually challenging bees, using both pinned‐specimen images and wing images. Hossain et al. (2021) compiled a balanced 11‐species bee dataset from iNaturalist and fine‐tuned multiple CNNs for species classification, reporting EfficientNet with NoisyStudent among the top‐performing models.

María‐Luisa and Jesús‐Ángel (2024) built and compared 4 customized CNN classifiers to detect *Vespa velutina* using a 4‐class dataset with extensive augmentation. They outlined a framework for real‐time image capture, transmission, and alerting to support rapid invasive‐hornet management.

##### 3.4.1.6 Classical Machine Learning

Rebelo et al. (2021) employed a traditional computer vision and classical machine learning pipeline. They proposed a fully automatic workflow that segments wing images, extracts skeletons and vein‐junction landmarks, and performs classification using k‐Nearest Neighbors (KNN) with modified Hausdorff distance, optionally incorporating color information and multiple segmentation variants. This study addresses the challenge of distinguishing species solely from bee wing images with highly similar morphology, using a dataset covering 48 species. The main innovation lies in the segmentation stage, where the combination of filtering operations and skeletonization enables strong performance without relying on deep learning. Despite using a simple KNN classifier, the method achieved high accuracy, with the best results obtained using combined segmentation strategies and color information, reaching 99.88% accuracy at the genus level and 96.90% at the species level. The results demonstrate the potential of using distinct morphological features for species identification, suggesting that future work could explore more advanced classifiers to further enhance performance.

##### 3.4.1.7 Vision Transformer

Although the study of Yoo et al. (2023) primarily focuses on honey bees, it includes in total 11 economically important bee species, including wild bees from genera such as *Xylocopa* and *Bombus*, and is therefore included in our review. The authors developed a hybrid architecture that applies a vision transformer encoder to features extracted by ResNet50. This design enables the use of a transformer architecture on relatively small datasets by using a convolutional backbone. After feature extraction, the proposed model flattens the ResNet feature maps and projects them into a linear embedding to form a sequence. Positional embeddings are then applied to divide the extracted features into patches. The transformer encoder consists of multiple stacked encoder blocks, each containing two main components: a self‐attention mechanism and a feed‐forward network. The self‐attention layer evaluates the relevance of each input embedding to the others and produces updated encodings, which are then processed by the feed‐forward network. The output encodings are passed to the next encoder block and finally fed into a fully connected layer for classification. Experimental results show that this hybrid structure outperforms state‐of‐the‐art CNNs and Vision Transformer models in classification accuracy.

#### 3.4.2 Habitat Study

Habitat‐level studies are less common, appearing in 7 publications (7/26), and they involve tasks such as mapping nesting sites or predicting hive locations. Among these 7 studies, 4 apply CNN‐based methods, 1 uses a tabular transformer, 1 employs a classical machine learning pipeline, and 2 involve other computer vision approaches such as remote sensing and object delineation.

##### 3.4.2.1 CNN‐Based Methods

Mueller and Buckner (2025) mapped ground‐nesting bee aggregations using UAV imaging and YOLOv5m at millimeter‐scale spatial resolution. This study enables rapid, repeatable population estimates and spatial clustering analyses for conservation. Knauer et al. (2022b) used Faster R‐CNN to detect cavities used by cavity‐nesting solitary bees. Kim et al. (2025) employed YOLOv5 in a small‐drone workflow that captures aerial imagery, detects wasp hives, and converts detections to GPS coordinates to estimate hive locations. Singha Roy et al. (2023) investigated insect microhabitats by analyzing the backgrounds of insect photographs. They employed a real‐time instance segmentation framework, YOLACT (Bolya et al. 2019), for background segmentation and ResNet‐50 for classification. This automated image‐background classification achieved an accuracy of over 97% relative to manual classification and suggests that habitat‐associated background features can influence species recognition in computer‐vision tasks.

##### 3.4.2.2 Classical Machine Learning Methods

Chieffallo et al. (2025) processed UAV imagery from 30 grasslands into orthomosaics labeled as flower or non‐flower. Multiple machine learning models, including Gradient Boosting Machine (GBM), Random Forest (RF), Support Vector Machine (SVM), and shallow Neural Network (NNET), were trained to classify pixels and estimate floral cover, which was then correlated with field‐measured bee abundance and diversity to provide a scalable proxy for pollinator monitoring.

##### 3.4.2.3 Other Computer Vision Methods

Guangnan and Zhenyou (2022) developed a multimodal pipeline that integrates text sentiment scoring, image classification, and spatial clustering. The dataset was collected from the Washington State Department of Agriculture (WSDA), which contains sighting reports with textual descriptions, images, geographic coordinates, timestamps, etc. For image‐based scoring, the authors used Baidu's cloud‐based animal recognition API to obtain a confidence score for *Vespa mandarinia*. This service is capable of classifying nearly 8000 animal species (Baidu Inc 2025), although its underlying model architecture is not publicly disclosed. The image score, together with text‐derived scores and spatial distance measures, is combined into a three‐layer artificial neural network (ANN) that outputs a probability representing the likelihood that a given report indicates a true *Vespa mandarinia* presence. The primary objective of the study is habitat‐related, focusing on environmental risk assessment and early warning of invasive *Vespa mandarinia*. The classification task is limited to filtering and providing supportive imagery information. For this reason, we categorize this publication under habitat‐level studies.

Neyns et al. (2025) combined remote sensing and deep learning to map willow (*Salix*) trees. They aimed to explore the relationship between *Salix* tree density and wild bee ( *Andrena vaga* ) nest aggregations. They used Light Detection and Ranging (LiDAR) data to perform tree crown segmentation, producing individual tree crown polygons across the image collection sites. For each segmented crown, multi‐temporal PlanetScope reflectance values and spectral indices were aggregated into per‐tree features. These features were further fed into a tabular transformer model for tree classification. The authors then mapped *Salix* trees across Braunschweig and analyzed the proximity and availability of these floral resources relative to *Andrena vaga* nest aggregations to inform urban pollinator conservation.

##### 3.4.2.4 Tabular Transformer

In the work of Neyns et al. (2025), a tabular transformer model called SAINT (Self‐Attention and Intersample Attention Transformer) (Somepalli et al. 2021) was used to classify trees as Salix versus non‐Salix based on multi‐temporal, multi‐spectral satellite data. As discussed in 3.4.2, the authors collected individual tree crown polygons across the study sites, and for each segmented crown, multi‐temporal reflectance values and spectral indices were aggregated into per‐tree feature vectors. As *Salix* exhibits seasonal reflectance patterns, information including the day of the year (DOY) and the spectral band was embedded within the reflectance sequence. Each tree crown was transformed into a 40 × 8 tabular representation with 40 time points and 8 spectral bands per time point. These embeddings were then used for time‐series classification. It should be noticed that this approach operates on numerical reflectance values derived from imagery rather than raw image pixels.

#### 3.4.3 Behavior Study

Behavioral studies include 3 publications that analyze foraging behavior, visitation patterns, colony activity, etc. Such studies typically rely on more diverse data types than individual images. For example, video frames are commonly used as inputs for tracking systems. They also involve more complex processing pipelines composed of multiple components, such as various classification tasks, time event recording, and cross‐frame behavioral comparisons.

Nevertheless, CNN‐based models appear consistently across all 3 studies as primary building blocks. In addition, tracking algorithms are widely adopted to follow objects across video frames and derive time‐dependent behavioral information. The centroid‐based tracking algorithm is one commonly used geometry‐based method. It links objects between frames by comparing the centroids of their detected bounding boxes. Zhang et al. (2021) introduced ByteTrack, a multi‐object tracking approach that enhances track continuity by associating both high‐confidence and low‐confidence detection boxes when matching them to existing trajectories. Rather than discarding low‐score detections, ByteTrack uses a two‐stage matching strategy, where high‐confidence detections are matched first and remaining tracks are then matched with low‐confidence ones. This design helps to recover objects with weaker detection, creating more stable and robust tracking across frames.

Knauer et al. (2022b) investigated the behavior of solitary bees. They studied bee foraging duration (the time between leaving and returning to the nest) and nest recognition ability (the number of incorrect cavities probed before locating the correct one). Their work has a particular focus on female bees by monitoring their flight activities and tracking the number of brood cells they build. To accomplish these tracking tasks, the authors employed a pipeline combining R‐CNN for bee detection, Faster R‐CNN for cavity detection, YOLOv3 for color‐tag identification, a custom Keras digit classifier for reading ID numbers, and a custom centroid‐based tracking algorithm to associate bee detections across video frames and reconstruct individual movement paths.

Varga‐Szilay et al. (2024) recorded five‐minute 5K video sequences of *Bombus terrestris* foraging on patches of *Lotus*, *Persicaria*, and *Trifolium*. They trained plant‐specific YOLOv5 models to detect bees on a frame‐by‐frame basis. They recorded visitation rates and time duration of individual bees on flowers versus off‐flowers. This approach enabled the analysis of plant‐specific attractiveness under uncontrolled field conditions. Their results showed that flower cover was the only factor significantly influencing bumblebee visitation, while plant species itself had no measurable effect on attractiveness.

Martínez et al. (2024) developed an autonomous monitoring station that records nest‐entrance traffic and applies YOLOv8 combined with ByteTrack to determine wasp movement direction. This system enables high‐throughput behavioral monitoring of yellowjacket (*Vespula germanica*) colonies, enriched with environmental covariates such as temperature and humidity. Using this setup, the authors analyzed ecological factors shaping colony growth, foraging dynamics, and the behavior of reproductive individuals.

### 3.5 Monitoring Platforms

#### 3.5.1 Mobile and Web Applications

Five publications reported the development of web or mobile applications.

Buschbacher et al. (2020) developed DeepABIS, a MobileNetV2‐based end‐to‐end system for image‐based identification of wild bee species. They provide both a mobile application (MobileABIS) and a web interface (CloudABIS) for wild bee species identification. MobileABIS, an Android application, allows users to identify bee species by taking a photo of a bee wing or selecting one from the photo gallery. The model is further optimized for Android deployment using TensorFlow Lite (n.d.). CloudABIS, a web application mirroring the mobile app's functionality, enables users to upload images via drag‐and‐drop and displays the inference results as a ranked top‐5 classification with class probabilities. The web application is built with the Laravel PHP framework (2022), which communicates with a Python‐based inference server running a TensorFlow backend.

Jeon et al. (2023) designed a portable, real‐time hornet detection and alert system integrated with a mobile application. Their system provides continuous apiary monitoring through RTSP video streaming and allows users to manage devices, configure alarm resend intervals, and review detection history directly within the app.

Shirali et al. (2024) developed a detector for parasitoid wasps and released a web application that supports lab‐quality images through a Hugging‐Face‐based (Face 2024) interface, providing both single‐image and batch processing capabilities (Both et al. 2023). Their models achieved high accuracy in genus‐level and sex‐level classification for *Diapriidae* and *Ismaridae* images. However, the authors also emphasized that only high‐quality laboratory images are suitable for reliable detection. Smartphone images, which often vary in resolution and lighting conditions, typically do not produce dependable results.

Chiranjeevi et al. (2024) developed InsectNet, a large‐scale web application for insect species identification trained on citizen‐science images spanning 2526 species. This system incorporates Out‐of‐Distribution (OOD) detection and conformal prediction to support more reliable real‐world deployment. The interface follows a minimalist, user‐friendly design in which users simply upload or capture an image containing an insect. The application then returns the predicted species and, when the model exhibits low confidence, warns the user that the input may be an OOD sample.

Based on similar citizen‐science principles but focused specifically on bumble bees, Spiesman et al. (2021) compiled a large bumble bee image dataset from citizen‐science sources. They trained and compared four CNN classifiers for 36 species and released the BeeMachine web application for public inference.

#### 3.5.2 Released Code

Eight publications (Bhuiyan et al. 2022; Chieffallo et al. 2025; Knauer et al. 2022b; Neyns et al. 2025; O'Shea‐Wheller et al. 2024; Spiesman et al. 2024; Srinivas et al. 2025; Varga‐Szilay et al. 2024) reported publicly available code or repositories. These publications are not specifically categorized as web or mobile applications. Instead, they typically provide scripts for experimentation, datasets, model training, evaluation, visualization, or other supplementary utilities.

Among these, the *BeeTracker* (Knauer et al. 2022b) stands out for its completeness, comprehensive documentation, and well‐developed multi‐functional monitoring. The BeeTracker software (Knauer et al. 2022a) consists of four trained deep‐learning networks. It is designed to perform multiple monitoring tasks, including detecting bees entering or leaving their nests, recognizing individual IDs on the bees' thoraxes, and identifying nest IDs based on their positions within the nesting unit. The BeeTracker is able to identify each nest associated with corresponding bees, enabling the observation of reproductive success. With a particular focus on female bees, the BeeTracker quantifies the number of cavities a female bee visits before locating her own nest as a proxy for nest recognition. It provides information on both the number and duration of foraging trips. When trained on eight videos, each recording 24 nesting females, the BeeTracker achieved a precision of 96% in correctly measuring these ecological factors. The BeeTracker can be adapted to a range of experimental setups by retraining it on an appropriate set of videos. It works as a promising tool for monitoring and assessing complex bee behaviors under laboratory, semi‐field, and field conditions.

#### 3.5.3 Hardware Setup

##### 3.5.3.1 Aerial Image Collection

Four studies reported the use of aircraft or unmanned aerial vehicles (UAVs) for aerial image collection. All these 4 publications focused on habitat‐level monitoring, where images were captured from above to study nesting sites and frequently visited flowers or trees.

In the work of Neyns et al. (2025), airborne LiDAR was used to generate tree segmentation and produce detailed 3D models of tree crowns. This data collection enabled researchers to map individual *Salix* trees using PlanetScope time‐series data and to analyze their ecological relationships with wild bee nesting‐site aggregations.

Aerial images contribute to generating precise nest coordinates, which can be used for spatial ecological analyses such as density estimation and clustering. Mueller and Buckner (2025) captured images of bee nests to study ground‐nesting bee aggregations. They used a DJI Air2S (consumer‐grade UAV) with a 20 MP, 1‐in. CMOS camera, flown at an altitude of 2 m above ground level, achieving a ground sampling distance of 0.6 mm/pixel. Automated flight planning was performed via QGIS Flight Planner, and the images were stitched into an orthomosaic using WebODM.

Similiarly, Chieffallo et al. (2025) used a DJI Matrice 210 RTK UAV equipped with an RGB Zenmuse X5 camera (16 MP, 17.3 × 13.0 mm sensor) and RTK GPS. Imagery was collected from 30 m above ground to capture high‐resolution RGB images of grassland transects, from which orthomosaics were constructed and subsequently labeled (flower versus non‐flower).

Focusing on wasp‐hive candidate locations, Kim et al. (2025) proposed a search strategy for identifying potential wasp‐hive sites. The proposed small‐drone‐based system uses 3DR Solo and a GoPro HERO4 Black camera to capture aerial images. They further use the captured images to construct a 3D map using ODM, render multi‐view images in QGIS, and subsequently detect hive candidates from these rendered views.

##### 3.5.3.2 Standalone Cameras

Five studies employed standalone cameras for image or video acquisition rather than relying solely on existing data sources. Shirali et al. (2024) used an Olympus E‐M10 paired with a Mitutoyo Plan Apo 5× microscope lens to capture laboratory‐quality images of parasitoid wasps. Jeon et al. (2023) acquired image data using GoPro Hero6 at the apiary of the National Institute of Agricultural Sciences (San Mateo, CA, USA). Varga‐Szilay et al. (2024) employed a GoPro Hero9 to record five‐minute 5K field videos of *Bombus terrestris* visiting different flowers. Spiesman et al. (2024) used a Nikon SMZ800N microscope with a Swiftcam SC503 camera and a 144‐LED ring light for capturing pinned bee specimens, and a Leica stereo microscope equipped with a Jenoptik ProgRes camera, as well as an Epson flatbed scanner, for wing images. Srinivas et al. (2025) developed a solar‐powered automated DSLR rig (Canon EOS 400D with a 70–300 mm Tamron lens, custom timer, and 18 V battery/solar panel) to build a field‐collected South Asian bee image dataset under fully uncontrolled field conditions.

##### 3.5.3.3 Monitoring Stations

Another 5 studies developed more complex monitoring stations equipped with components such as cameras, sensors, embedded processors, and communication modules. These monitoring stations are designed for combined tasks, including individual classification, tracking movement paths, recording temporal information, monitoring changes within nesting cells, etc.

Jeon et al. (2023) designed a portable, real‐time hornet detection and alert system for apiaries, integrating YOLOv5s on an embedded GPU with RTSP video streaming. This system performs *Vespa velutina* detection every 10 frames and sends alerts with captured images to the user. It consists of an on‐site image analysis and communication module, a mobile application for user interaction, and a server that manages communication between the two.

Knauer et al. (2022b) proposed the BeeTracker software, which has been discussed in Section 3.5.2. Its hardware relies on a video‐based monitoring setup: digital video cameras positioned 1 m in front of nesting units to capture frontal views of hive cavities; nesting units constructed from layered wooden boards containing 10 cavities per layer; and large flight cages (54 m^2^) providing adequate floral resources. Individual bees are marked with colored digit ID tags to enable automated identification within the recorded videos.

Martínez et al. (2024) developed a monitoring station composed of an illuminated channel and a fixed camera to capture wasp activity. The station (23 cm tall, 19 cm wide, 16 cm deep) included five 3D‐printed components fabricated in black filament (polylactic acid), which formed an acrylic walkway through which wasps enter or exit while being recorded. The system incorporated an NVIDIA Jetson Nano Developer Kit B‐01 with a 500 GB mini‐SD card and a Sony IMX219‐130 wide‐angle digital camera positioned 10.8 cm above the walkway. Custom post‐processing software was implemented to determine movement direction and caste of the recorded individuals.

O'Shea‐Wheller et al. (2024) built an integrated prototype system combining software, hardware, and a bait‐station. Each bait station used a Dragon Touch Vision 11080p camera suspended 210 mm above a featureless detection board and enclosed with an opaque baffle to minimize background variation. A Raspberry Pi 4 with camera and power supply enabled real‐time video capture and analysis, transmitting candidate detections to a paired computer via local Wi‐Fi. These stations were used to collect extensive image datasets of wasps and other insects.

Braga and Madureira (2020) designed a Decision Support System (DSS) that continuously schedules multi‐camera inputs. In their system, the cameras act as real‐time, GPS‐enabled visual sensors. The image stream is then fed into a detection pipeline controlled by a scheduler and linked to an alert mechanism that records, localizes, and reports sightings of Asian hornets.

### 3.6 Existing Datasets

Manual data collection is time‐consuming and often requires substantial resources such as advanced cameras, laboratory equipment, or monitoring stations, as discussed in Section 3.5.3. Existing datasets play a crucial role in many of the reviewed studies. In total, ten (10/26) of the selected publications rely on publicly available or previously collected datasets rather than acquiring new data themselves. These datasets typically do not contain images from a single specific location, and they normally include globally sourced images.

#### 3.6.1 iNaturalist

##### 3.6.1.1 Overview

iNaturalist (n.d.) is a global citizen science observation platform that allows people to map observations of organisms and share photographic records of biodiversity across the globe. It provides users with both web and mobile applications. Each observation consists of a date, location, image, and labels containing the name of the species present in the image. Until November 2025, there have been 286,457,433 observations, 542,748 species, 465,842 identifiers, and 3,971,990 observers (iNaturalist, n.d.). If the community reaches a consensus on the taxa in an observation, a “research‐grade” label is applied to it (Van Horn et al. 2017). iNaturalist makes an archive of “research‐grade” observations, which is available via the Global Biodiversity Information Facility (GBIF) (iNaturalist Contributors 2025).

One of the most widely used subsets from the iNaturalist database is iNat2017 (Van Horn et al. 2017). The iNat2017 dataset is designed for species classification and detection. It was first introduced in 2017 and comprised images and labels from the iNaturalist GBIF archive. iNat2017 contains over 5089 taxa from 13 super‐classes, with 579,184 training images, 95,986 validation images, 182,707 test images, and over 560,000 manually created bounding boxes. The goal of iNat2017 is to advance the state‐of‐the‐art in image classification and detection for “in the wild” data featuring large numbers of imbalanced, fine‐grained categories. The 13 super‐classes include Plantae, Insecta, Mammalia, Fungi, etc. The super‐class *Insecta* contains 1021 species, 100,479 training images, 18,076 validation images, and 125,679 bounding boxes.

Popular subsets from the iNaturalist include the iNaturalist2018 (2018), with a total of 8142 species in the dataset, 437,513 training images, and 24,426 validation images. Each image has one ground truth label. The iNat Challenge 2019 (2019) dataset is a relatively small dataset, containing 1010 species with a combined training and validation set of 268,243 images that were collected and verified by multiple users from iNaturalist. iNaturalist2021 (Grant Van Horn and Aodha 2021), which contains a total of 10,000 species. The full training dataset includes nearly 2.7 M images. To make the dataset more accessible, a “mini” training dataset has been created with 50 examples per species, for a total of 500 K images. It covers 11 super‐classes, spanning 2526 insect species.

##### 3.6.1.2 Customized Subsets From iNaturalist

Among the reviewed publications, six studies (5/26) rely on images from iNaturalist observations, and they use customized subsets from iNaturalist. The authors selectively choose images from specific genera or taxonomic groups that align with their ecological objectives. Yoo et al. (2023) conducted an exhaustive literature search and identified a list of the world's most important bee genera from iNaturalist. Bhuiyan et al. (2022) used 6332 images from the iNaturalist platform, containing bumble bee versus other insects. Only images marked with Research Grade were downloaded. They focused on 25 species that occur frequently in Germany. Chiranjeevi et al. (2024) trained on 2526 insect species categories from the *Insecta* class using the 2017 iNaturalist dataset. María‐Luisa and Jesús‐Ángel (2024) used 1200 images of *Vespa velutina* from iNaturalist. Hossain et al. (2021) sourced an image dataset of 11 species of bumble bee (*Bombus*), honey bee (*Apis*), and carpenter bee (*Xylocopa*) from iNaturalist.

#### 3.6.2 Other Datasets

Three publications (3/27) used private datasets. Among them, two works from Buschbacher et al. (2020), Buschbacher and Steinhage (2020) used material collected in the 1990s and early 2000s at the former Institute of Agricultural Zoology and Bee Biology at the University of Bonn. Specimens were collected in Germany, the United States, Brazil, and China. Rebelo et al. (2021) used wing images from the laboratory. This dataset was provided by a co‐author at the University of São Paulo.

Three publications (2/27) involve images from online platforms. María‐Luisa and Jesús‐Ángel (2024) used non‐Vespa insect images from the image platform Freepik (2025). Braga and Madureira (2020) used web‐sourced live streams and videos.

## 4 Conclusion

### 4.1 Limitations

#### 4.1.1 Research Focus

**FIGURE 8:**
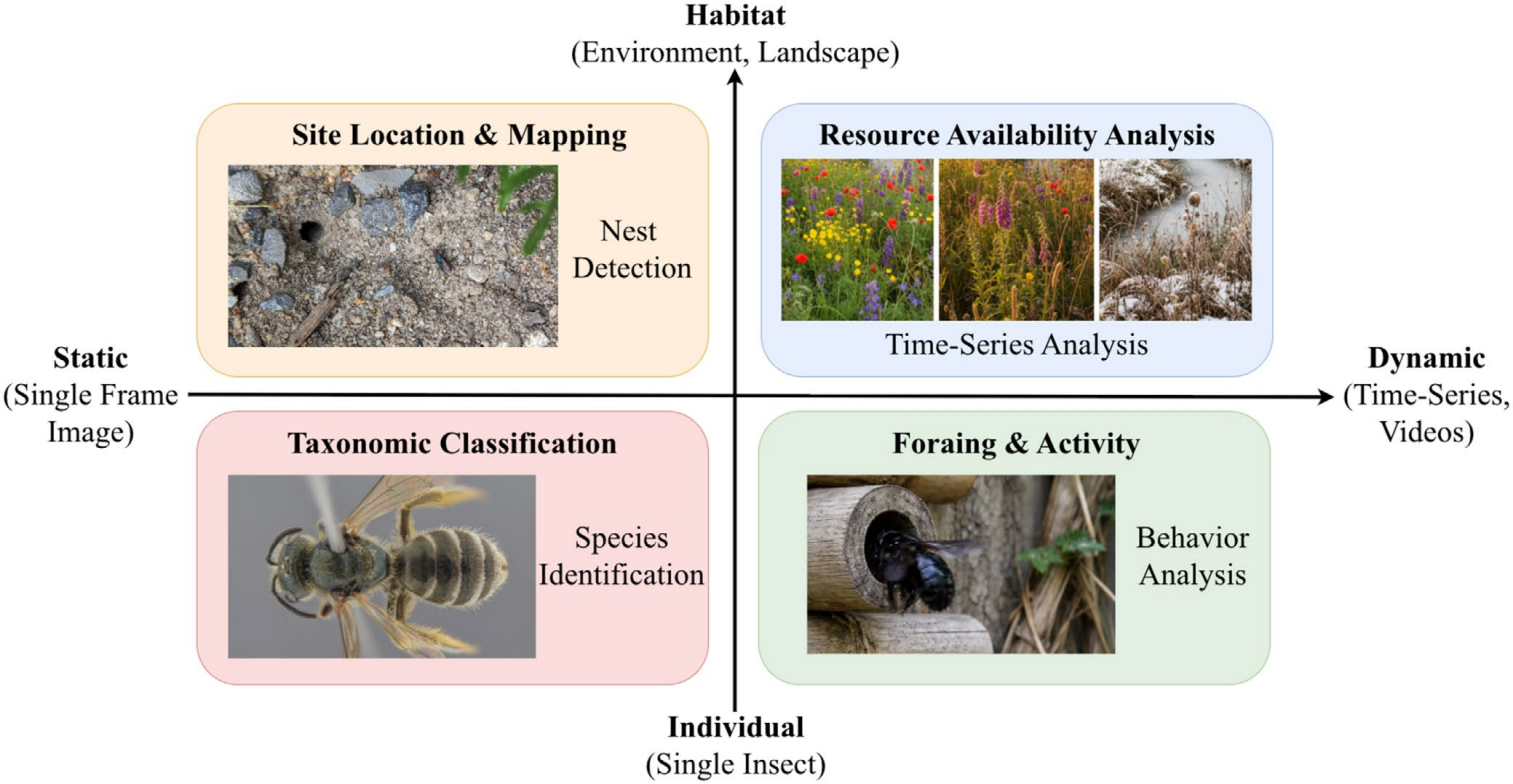
Categorization of research gaps in ecological analysis across temporal (*x*‐axis: *Static* vs. *Dynamic*) and spatial (*y*‐axis: *Individual* vs. *Habitat*) dimensions.

To visually identify current research gaps and clustering within this review, Figure 8 categorizes the reviewed literature into a matrix defined by the spatial scale of the subject and the temporal dimension of the data. The majority of existing publications cluster in the bottom‐left quadrant (*Static*/*Individual*), focusing on taxonomic classification using data sources like *iNaturalist*. The limited focus of research scopes on the upper and right‐hand quadrants highlights significant opportunities for future research in dynamic behavioral tracking and habitat‐level monitoring.

Based on the findings of this review, research on resource availability analysis (top‐right quadrant in Figure 8), which involves dynamic habitat studies across time, remains limited. Existing publications from ecological phenology, such as (Katal et al. 2025; Rzanny et al. 2024), focus on interactions between plants and their environment by analyzing plant distributions across large spatial and temporal scales. These studies provide us insights into potential future research on interactions between habitats and wild insects across time. In this context, computer vision methods could be applied in future work.

#### 4.1.2 Species Coverage

*Hymenoptera* is among the most diverse and abundant insect orders and plays essential ecological roles as a food source, pest controller, and pollinator (Peters et al. 2017). Despite its importance, much of its diversity and biology remains poorly understood. *Hymenoptera* includes more than 153,000 described species and may comprise up to one million undescribed extant species (Peters et al. 2017). Although some well‐known wild bee groups, such as bumblebees (*Bombus* spp.), attract comparatively greater interest in ecological research, broader awareness of wild bee diversity remains limited (Ghisbain 2021). At the same time, current bee research remains largely restricted to particular regions or site‐based studies (Orr et al. 2021). Even in relatively well‐studied regions such as North America, many species may still remain undescribed (Dorey et al. 2026). This is particularly true in larger taxonomic groups, where species are often considered together rather than studied individually. As a result, improved identifiability and more accurate taxonomic decisions for undescribed species are still needed (Dorey et al. 2026).

This limitation is also reflected in the results of our review. Most computer vision methods continue to focus on common and well‐known genera, such as the wild bee genus *Bombus*, whereas rare species receive little or no attention. This tendency is even more pronounced in wasp‐related studies, where only a few common species from *Vespa* and *Vespula* are included, while other genera are entirely overlooked. Overall, current studies appear to follow two main practical directions: (1) they emphasize the ecological importance of wild bees, but still rely on common species as representative examples; and (2) they aim to develop solutions for the protection of domesticated bees or the control of wasp populations, and therefore typically include only a few common wasp species relevant to these objectives. As a result, rare species are largely absent from reviewed publications and receive little ecological attention. Consequently, ecological discussions are often superficial or limited to very specific questions, as most emphasis is placed on the application of computer vision methods rather than generating new ecological insights. In contrast, studies specifically dedicated to the observation, discovery, and investigation of rare species remain scarce.

#### 4.1.3 Computer Vision Methods

The computer vision methods reviewed are all based on supervised learning pipelines. Most of them rely on CNNs, while hybrid CNN‐transformer architectures also appear in a limited number of recent publications. As these methods learn directly from labeled samples, their performance depends strongly on the quality and consistency of the underlying labels (Bhatt et al. 2021).

Supervised deep learning methods are inherently label‐intensive, and their successful application to wild bee and wasp monitoring depends critically on the availability of high‐quality labels, as well as sufficiently large and diverse image datasets. First, taxonomic identification in these groups is often difficult even for experts, as species discrimination depends on subtle morphological characters that are not always visible in photographs (Colgan et al. 2024). Consequently, image resolution, uncertain identifications, and inconsistencies in image context can directly influence both model training and evaluation (Suzuki‐Ohno et al. 2022). Second, current methods are strongly dependent on large volumes of labeled data and extensive image collections. As noted above, many rare species remain undescribed, and the long‐term collection of broad‐scale information across a wide range of species is prohibitively expensive (Feldman et al. 2021). As a result, around one‐third of the reviewed publications (9/26) relied on existing global citizen science platforms rather than on self‐collected datasets.

Although a few publications acknowledge these limitations, their discussion remains largely general and does not offer a comprehensive analysis of methodological strategies for improving data efficiency. Addressing this gap will require future work to move beyond standard supervised pipelines and to explicitly investigate approaches that reduce reliance on large labeled datasets. One promising direction is the more systematic use of advanced augmentation strategies. Augmentation methods tailored to related ecological vision tasks have already been explored in the broader literature, including synthetic image generation and manipulation‐based augmentation for small insects in field images and insect trap images (Nitin et al. 2023; Saradopoulos et al. 2024; Suto 2024). Several recent studies highlight the strong potential of emerging model architectures for improving data efficiency. For example, semi‐supervised learning has shown promise in automated insect and pest monitoring (Gomez‐Zamanillo et al. 2025; Rustia et al. 2021). In addition, related approaches such as self‐supervised pretraining and zero‐shot detection have been successfully applied to fine‐grained insect species identification, offering new perspectives to overcome the limitations of traditional CNN‐based methods (Chiranjeevi et al. 2024; Feuer et al. 2024).

Additionally, to avoid relying solely on phenotype‐based identification, computer vision approaches could be integrated with taxonomic expertise and genetic methods such as DNA barcoding to improve species verification and support the construction of more reliable training datasets (Høye et al. 2021). DNA barcoding is a molecular identification method that uses a short standardized DNA sequence to assign specimens to species through comparison with a reference library (Hebert et al. 2003). For example, in *Hymenoptera*, barcoding can be applied shortly after trap‐nest collection by using larvae or dead specimens as DNA sources (Turčinavičienė et al. 2014). A substantial foundation for DNA barcoding has already been established, with reference libraries covering a large number of species. Research has shown that DNA barcoding records are largely congruent with traditional taxonomy and can support large‐scale and reliable identification of wild bees and wasps (Schmid‐Egger et al. 2024, 2018; Schmidt et al. 2015). Beyond species identification, DNA barcoding can also contribute to research on bee behavior and habitat. For instance, DNA‐based methods create datasets and improve floral resource characterization, such as through the molecular analysis of pollen, which may be useful for beekeepers seeking honeybee products with specific nutritional or therapeutic properties (Galimberti et al. 2014). Similarly, DNA‐based approaches have been used to address the challenge of identifying food resources used by nesting bees and wasps, including the reconstruction of quantitative three‐ and four‐trophic interaction networks in apoid wasp families (Fornoff et al. 2023). In our review, however, only one study explicitly incorporated DNA‐based identification, suggesting that this direction remains largely underexplored.

#### 4.1.4 Data Source

The datasets used in the reviewed publications can be grouped into three categories: (1) Self‐collected datasets created for specific problems in each publication. (2) Public datasets, subsets derived from public datasets, and Internet images. (3) Private datasets that are not publicly accessible. A key limitation is the source of public datasets, as all publications using public data rely exclusively on iNaturalist images. A common practice from the reviewed publications is to construct customized datasets by selecting specific taxa from the iNaturalist database. Although other open ecological datasets exist, such as those provided by platforms like Observation.org (n.d.) or Kaggle (n.d.), none of the reviewed publications made use of them.

#### 4.1.5 Application

There is a clear gap in the development of practical applications, and several major limitations can be identified. (1) Although many reviewed publications report released code, most remain at early or incomplete stages. Many repositories provide only basic components such as datasets, training scripts, and evaluation scripts, without delivering a fully functional, ready‐to‐use application. (2) Some studies describe the development of web or mobile applications, but these typically remain as demos that are not officially released. Their functionalities are relatively limited to one or two basic functions, such as detection or classification. (3) Almost all existing applications focus on a narrow set of bee or wasp taxa within restricted environmental contexts. They lack comprehensive species coverage, especially on rare species. They also do not support broader cross‐geographical information and analysis.

### 4.2 Future Work

Future work should focus on two main objectives: (1) Explore and make full use of existing data sources that have not yet been used in the reviewed publications. (2) Develop multi‐functional platforms that incorporate multiple ecological focuses by combining existing functionalities identified in the reviewed publications, as well as integrating new monitoring tasks that are not covered in these publications.

#### 4.2.1 Current Foundations

There are some strong examples of data sources. For instance, Observation.org (n.d.) is a global citizen‐science biodiversity platform where users can record, share, and verify observations of plants, animals, and other species. It serves as a valuable data source, similar to iNaturalist, and covers a wide range of species within the order of *Hymenoptera*, including bees and wasps. Its primary dataset contains more than 250 million observations and grows by around 35 million observations per year, contributed by over 450,000 users. Data quality is maintained through the combined efforts of more than 1000 domain experts supported by smart technology and artificial intelligence (Observation.org, n.d.).

Some existing tools also offer useful insights into the future development of more comprehensive monitoring platforms. The BeeMachine (n.d.) provides a solid example of potential directions for wild bee monitoring. As discussed in section 3.5.1, the publication by Spiesman et al. (2021) presented the prototype version of BeeMachine, which enabled users to identify bumble bees. At the time of publication in 2021, it was still in an early stage of development. In recent years, BeeMachine has been updated frequently to improve usability and accuracy while expanding the number of species included in its classification model. It now offers Android, iOS, and web versions, and is capable of recognizing 354 types of flower‐visiting insects. This includes 221 bee species, 128 bee genera, and additional groups such as wasps, flies, hoverflies, beetles, butterflies, and moths. In 2025, BeeMachine introduced an enhanced feature that combines image recognition with spatial and temporal data, allowing the system to use both the observed image and the corresponding location and time to generate more accurate species predictions (n.d.).

The Beexplainable (Chiaburu 2025) also provides a good example. This repository first introduces a dataset derived from iNaturalist. It provides scripts for data acquisition, which download photographs of bees from iNaturalist, search the index for specific taxa, and perform duplication checking and removal. It also includes tools for annotation and segmentation of distinct bee parts, such as *head*, *thorax*, and *abdomen* (Chiaburu et al. 2022). In addition to data collection, the authors incorporated a detector based on ResNet50, initialized with a backbone pretrained on the iNatChallenge 2021 (2021) and implemented species classification on top. The full dataset was used for training and cross‐validation, while the mini dataset was reserved as a test set. With a focus on model explainability, the authors supervised the monitoring procedure and investigated what factors contributed to the model's predictions using Explainable Artificial Intelligence (XAI) methods. Libraries such as tf‐explain (Meudec 2021) and iNNvestigate (Alber et al. 2019) for generating explanations, and Quantus (Hedström et al. 2023) are incorporated for evaluation.

Another example is MapBee (n.d.), a collaborative project between TU Ilmenau and the Julius Kühn‐Institute in Germany. MapBee is a specialized web application designed to systematically map and document wild bee observations across different geographical regions. It provides a central platform for recording and evaluating sightings, and it predicts both the species and the gender of a bee based on the images uploaded by users.

#### 4.2.2 Future Directions

Eventually, we propose a more comprehensive monitoring framework for wild bee and wasp monitoring. In addition to allowing users to upload observations, it should also incorporate the following perspectives. (1) Refine and expand the use of datasets, considering wild bees, wasps, and common mimics, with genus‐ and species‐level classification and detection as basic functions. (2) Rare wild bee and wasp taxa should receive considerably more attention during training. (3) Compare different model structures and introduce explainability, such as highlighting image regions or features that influence model decisions. Geographic and temporal factors, as implemented in BeeMachine (n.d.), can also be incorporated to improve prediction accuracy. (4) Provide users with scientific information based on their locations, including species glossaries and conservation actions. For example, suggesting specific plants suitable for supporting particular bee species in home gardens. (5) Summarize the sightings from users across time, and study bee aggregation and other behaviors over different months and seasons.

## Author Contributions

**Chenchang Liu:** data curation (lead), investigation (lead), visualization (lead), writing – original draft (lead), writing – review and editing (lead). **Patrick Mäder:** funding acquisition (lead), project administration (lead), supervision (lead), writing – review and editing (supporting). **Marco Seeland:** conceptualization (lead), formal analysis (lead), investigation (supporting), supervision (lead), visualization (supporting), writing – review and editing (lead).

## Funding

C.L., P.M., and M.S. are supported by the Federal Agency for Nature Conservation (BfN) through the project “BeesUp” (Grant Number: 3520685B29).

## Conflicts of Interest

The authors declare no conflicts of interest.

## Supporting information


**Table S1:** Overview of all reviewed publications.

## Data Availability

This article is a review and does not involve the generation or use of new datasets. All information analyzed is based on previously published studies cited in the manuscript.
