# Supplementary material for: Computer Vision for Monitoring Wild Bees and Wasps: A Structured Literature Review
Source: Ecol Evol. 2026 Jun 30;16(7):e73794. doi: 10.1002/ece3.73794 (PMC13318512; doi:10.1002/ece3.73794)
Supplement: Supplementary file 1 — Table S1: Overview of all reviewed publications. [file ECE3-16-e73794-s001.pdf]

ORIGINAL ARTICLE
Journal Section

Supplementary Material

Chenchang Liu[1] | Patrick Mäder[1,2,3] | Marco Seeland[1*]

[1]Department of Computer Science and Automation, Technische Universität Ilmenau, Ilmenau, Thuringia, 98693, Germany

[2]Faculty of Biological Sciences, Friedrich-Schiller-Universität Jena, Jena, Thuringia, 07745, Germany

[3]German Centre for Integrative Biodiversity Research (iDiv), Leipzig, Saxony, 04103, Germany

Correspondence
Marco Seeland
Department of Computer Science and Automation, Technische Universität Ilmenau, Ilmenau, Thuringia, 98693, Germany
Email: marco.seeland@tu-ilmenau.de

Funding information
C.L., P.M., and M.S., Grant/Award Number: 3520685A29, Federal Agency for Nature Conservation (BfN), project “BeesUp”

Wild bees and wasps are vital to ecosystems, yet large-scale monitoring of individual insects as well as their habitats and behaviors requires expert knowledge and is very labour-intensive. Recent advances in computer vision offer both methodological solutions and practical applications for these tasks. This review systematically surveys state-of-the-art literature (2020-2026) on computer-vision-based monitoring of wild bees and wasps. We compare the primary monitoring tasks, such as individual detection and classification, habitat observation, and assessment of insect behavior, and analyze how specific computer vision techniques contribute to each. By examining the datasets used in the reviewed studies, we further categorize dataset types and collection strategies to inform future image acquisition. In addition, we draw insights from widely used public resources (e.g., iNaturalist, Observation) regarding their strengths and limitations for this domain. We then examine how hardware and software are integrated in these studies, and review released repositories, existing applications, and the design of more complex, multifunctional monitoring stations. These analyses provide guidance for benchmarking and future deployment with existing datasets and monitoring tools. We eventually propose the design of building more comprehensive and efficient automatic monitoring systems for wild bees and wasps.

KEYWORDS

ecological monitoring, nature conservation, computer vision, deep learning

1 Reviewed Publications

TABLE 1 Overview of all reviewed publications.

Authors	Year	Title	Focus	Region	Data
Buschbacher *et al.* [1]	2020	Image-based species identification of wild bees using convolutional neural networks	Individual classification	Germany	Bee
Buschbacher *et al.* [2]	2020	On the extraction and relevance ranking of visual morphological traits for taxon identification	Individual classification with explainability of morphological traits	Germany	Bee
Kelley *et al.* [3]	2021	Honey sources: neural network approach to bee species classification	Individual classification	United States	Bee
Yoo *et al.* [4]	2023	BeeNet: An End-To-End Deep Network For Bee Surveillance	Individual classification and health monitoring	Australia	Bee
Bhuiyan *et al.* [5]	2022	Artificial intelligence versus natural selection: Using computer vision techniques to classify bees and bee mimics	Individual classification and behavior study on mimicry	United States	Bee, bee mimics
Knauer *et al.* [6]	2022	Bee Tracker: an open-source machine learning-based video analysis software for the assessment of nesting and foraging performance of cavity-nesting solitary bees	Individual detection and behavior study	Switzerland	Bee
Varga-Szilay *et al.* [7]	2024	Flower Visitation through the Lens: Exploring the Foraging Behaviour of *Bombus terrestris* with a Computer Vision-Based Application	Behavior study	Hungary	Bee
Spiesman *et al.* [8]	2024	Deep learning for identifying bee species from images of wings and pinned specimens	Individual classification	United States	Bee
Srinivas *et al.* [9]	2025	Image-based South Asian bee species identification: a machine learning approach	Individual classification	India	Bee

Continued on next page

Authors	Year	Title	Focus	Region	Data
Spiesman *et al.* [10]	2021	Assessing the potential for deep learning and computer vision to identify bumble bee species from images	Individual classification	United States	Bee
Hossain *et al.* [11]	2021	A Machine Learning Based Approach to Study Morphological Features of Bees	Individual classification with morphological feature explanation	Australia	Bee
Neyns *et al.* [12]	2025	Deep learning based mapping of bee-friendly trees through remote sensing: A novel approach to enhance pollinator conservation	Habitat study	Belgium	Bee habitat
Chieffallo *et al.* [13]	2025	Machine learning for biodiversity: UAV-based flower detection as an indirect proxy for bee abundance	Habitat study	Italy	Bee habitat
Mueller and Buckner [14]	2025	UAV-based Remote Sensing of Bee Nesting Aggregations with Computer Vision for Object Detection	Habitat study	United States	Bee habitat
Kwon *et al.* [15]	2024	Proposal of an advanced YOLOX model for real-time detection of *Vespa* hornets (Hymenoptera: Vespidae), key pests of honey bees	Individual detection	Republic of Korea	Wasp
Guangnan and Zhenyou [16]	2022	A framework for predicting the location of *Vespa mandarinia* nests via deep learning approach	Habitat study	China	Wasp
Jeon *et al.* [17]	2023	Deep Learning-Based Portable Image Analysis System for Real-Time Detection of *Vespa velutina*	Health monitoring	Republic of Korea	Wasp
María-Luisa and Jesús-Ángel [18]	2024	Deep Learning for *Vespa velutina* Detection	Individual classification	Spain	Wasp
Singha Roy *et al.* [19]	2023	Image background assessment as a novel technique for insect microhabitat identification	Habitat study	Australia	Bee and wasp habitat
Kim *et al.* [20]	2025	Wasp-Hive Candidate Site Search System Using a Small Drone	Habitat study	Republic of Korea	Wasp

Continued on next page

Authors	Year	Title	Focus	Region	Data
Martínez *et al.* [21]	2024	Advancing social insect research through the development of an automated yellowjacket nest activity monitoring station using deep learning	Behavior study	Argentina	Wasp
O'Shea-Wheller *et al.* [22]	2024	VespAI: a deep learning-based system for the detection of invasive hornets	Invasive species early detection	United Kingdom	Wasp
Shirali *et al.* [23]	2024	Image-based recognition of parasitoid wasps using advanced neural networks	Individual classification with outlier detection	Germany	Wasp
Braga and Madureira [24]	2020	Towards a decision support system for the automatic detection of Asian hornets and removal planning	Invasive species detection	Portugal	Bee and wasp
Chiranjeevi *et al.* [25]	2024	InsectNet: Real-time identification of insects using an end-to-end machine learning pipeline	Individual classification	United States	Multiple insect taxa
Rebelo *et al.* [26]	2021	A fully automatic classification of bee species from wing images	Individual classification	Brazil	Bee

2 Summary of Reviewed Computer Vision Methods

- **Classification and Detection of Individual Insects**
  - **ResNet family.** ResNet is the most frequently used architecture in this task category. It is mainly applied to species classification, especially when robust feature extraction is needed for visually challenging taxa. Reported variants include ResNet50, ResNet101, Wide-ResNet101, ResNet101e, TResNet, CSPResNet50, and ResNetv2. In the reviewed studies, ResNet-based models were used for tasks such as species classification, mimicry identification. In some cases, ResNet also served as a backbone for more complex pipelines, such as hybrid CNN-transformer models.
  - **YOLO family.** YOLO models are widely used for real-time insect detection and monitoring. Reported variants include YOLOX, YOLOv3, YOLOv5s, and YOLOv8. These models are mainly used when efficient localization is required, for example in hornet detection, parasitoid wasp recognition, nest traffic monitoring, pinned-specimen pre-cropping, and bee-tag reading systems. Theses studies emphasize YOLO's suitability for small-object detection and deployable real-time monitoring systems, especially in field or apiary settings.
  - **R-CNN family.** Region-based CNN models, including Faster R-CNN and Mask R-CNN, are used for detection tasks that require more explicit region proposal mechanisms. In the reviewed publications, Faster R-CNN was used for detecting bees in raw, unprocessed photographs and in bee-tracking pipelines, while Mask R-CNN was used for wasp detection with instance-level localization. These models are suited to problems where

localization quality is important and where the pipeline must deal with cluttered or low-quality input images.

- **MobileNet family.** MobileNet, especially MobileNetV2, appears in lightweight classification systems for species recognition. It is mainly used in applications where compact models and computational efficiency are important. In the reviewed studies, MobileNetV2 was applied to wing-based wild bee species identification, explanation of relevant morphological regions, and outdoor species classification under uncontrolled imaging conditions.
- **VGG.** VGG16 was used for bee species classification and for distinguishing bees from visually similar bee and wasp mimics. Its role in the reviewed studies is mainly as a benchmark or comparison architecture rather than as the dominant model family.
- **Inception.** InceptionV3 was tested in species-classification studies, including South Asian bee classification and bumblebee identification.
- **EfficientNet.** EfficientNet is a family of models that scale depth, width, and resolution in a balanced way using a compound coefficient. It was applied to both pinned-specimen and wing-image datasets, and one study reported EfficientNet with NoisyStudent among the top-performing benchmark models.
- **Other CNN architectures.** Additional CNN-based models include RegNetY-32 in a self-supervised insect-classification framework and customized CNN classifiers for *Vespa velutina* detection. These models appear less frequently but show that the methodological landscape extends beyond the main model families.
- **Hybrid CNN–Vision Transformer model.** One study combines a ResNet50 backbone with a vision transformer encoder. In this design, the CNN first extracts image features, which are then converted into a sequence representation for transformer-based processing. This hybrid model is presented as a way to exploit transformer-based feature interactions on relatively small datasets.
- **Classical computer vision and machine learning pipeline.** One publication applies a traditional pipeline consisting of wing segmentation, skeleton extraction, vein-junction landmark detection, and KNN classification with modified Hausdorff distance. This approach is used for morphology-based bee species identification from wing images and demonstrates that carefully designed classical methods can still achieve high performance for highly structured taxonomic traits.

- **Habitat Study**
  - **YOLO family.** YOLO-based models are used in several habitat-oriented studies for spatial detection tasks. YOLOv5m was used to map ground-nesting bee aggregations from UAV imagery at very high spatial resolution. YOLOv5 was also used in a drone-based workflow for detecting wasp hives and converting detections into GPS-based location estimates. These applications show that YOLO is useful not only for individual insect detection but also for habitat-scale monitoring tasks based on aerial or spatial imagery.
  - **Faster R-CNN.** Faster R-CNN was used in a habitat-related context to detect cavities used by cavity-nesting solitary bees. In this setting, the model supports identifying nesting structures rather than directly classifying the insects themselves.
  - **YOLACT + ResNet-50.** One study used YOLACT for real-time instance segmentation and ResNet-50 for classification when analyzing insect microhabitats from the backgrounds of insect photographs. The goal was not only to recognize insects but also to classify the habitat context visible in the image background.
  - **Classical machine learning models.** One habitat-level study used classical machine learning rather than deep learning to estimate floral cover from UAV-derived orthomosaics. The tested models included Gradient Boosting Machine (GBM), Random Forest (RF), Support Vector Machine (SVM), and a shallow neural network (NNET). These models were used for flower versus non-flower classification, and the resulting floral-cover estimates were linked to field measurements of bee abundance and diversity.
  - **Tabular transformer (SAINT).** A tabular transformer model, SAINT, was used to classify trees as *Salix* versus

non-*Salix* based on multi-temporal spectral reflectance features derived from remote sensing imagery. In this case, the model works on structured numerical features aggregated from segmented tree crowns.

- **Behavior Study**
  - **CNN-based detection and classification models.** All 3 behavioral studies rely on CNN-based models from YOLO and R-CNN families as core components. In this category, CNNs are used as building blocks for detecting insects, classifying activity states, or identifying relevant events from image or video data. Compared with individual-level classification tasks, these CNNs are usually embedded within larger temporal-analysis pipelines rather than being the sole analytical model.
  - **Tracking algorithms.** Tracking is a key component of behavior studies as these tasks depend on temporal information rather than isolated images. Tracking methods are used to follow insects across frames and derive behavioral measures such as foraging activity, visitation patterns, or colony traffic. Tracking methods range from simple centroid-based approaches, which associate objects by the positions of their bounding-box centers, to more advanced methods such as ByteTrack, which improves trajectory continuity by matching both high-confidence and low-confidence detections across frames.

references

[1] Buschbacher K, Ahrens D, Espeland M, Steinhage V. Image-based species identification of wild bees using convolutional neural networks. Ecological Informatics 2020 Jan;55:101017. http://dx.doi.org/10.1016/j.ecoinf.2019.101017.

[2] Buschbacher K, Steinhage V. On the extraction and relevance ranking of visual morphological traits for taxon identification. Ecological Informatics 2020 Nov;60:101138. http://dx.doi.org/10.1016/j.ecoinf.2020.101138.

[3] Kelley W, Valova I, Bell D, Ameh O, Bader J. Honey sources: neural network approach to bee species classification. Procedia Computer Science 2021;192:650–657. http://dx.doi.org/10.1016/j.procs.2021.08.067.

[4] Yoo J, Siddiqua R, Liu X, Ahmed KA, Hossain MZ. BeeNet: An End-To-End Deep Network For Bee Surveillance. Procedia Computer Science 2023;222:415–424. http://dx.doi.org/10.1016/j.procs.2023.08.180.

[5] Bhuiyan T, Carney RM, Chellappan S. Artificial intelligence versus natural selection: Using computer vision techniques to classify bees and bee mimics. iScience 2022 Sep;25(9):104924. http://dx.doi.org/10.1016/j.isci.2022.104924.

[6] Knauer AC, Gallmann J, Albrecht M. Bee Tracker—an open-source machine learning-based video analysis software for the assessment of nesting and foraging performance of cavity-nesting solitary bees. Ecology and Evolution 2022 Mar;12(3). http://dx.doi.org/10.1002/ece3.8575.

[7] Varga-Szilay Z, Szövényi G, Pozsgai G. Flower Visitation through the Lens: Exploring the Foraging Behaviour of Bombus terrestris with a Computer Vision-Based Application. Insects 2024 Sep;15(9):729. http://dx.doi.org/10.3390/insects15090729.

[8] Spiesman BJ, Gratton C, Gratton E, Hines H. Deep learning for identifying bee species from images of wings and pinned specimens. PLOS ONE 2024 May;19(5):e0303383. http://dx.doi.org/10.1371/journal.pone.0303383.

[9] Srinivas CL, Sreerag M, Mukherjee R, Devy SM, Mishra S, Deb R. Image-based South Asian bee species identification: a machine learning approach. Journal of Insect Conservation 2025 Jul;29(4). http://dx.doi.org/10.1007/s10841-025-00691-7.

[10] Spiesman BJ, Gratton C, Hatfield RG, Hsu WH, Jepsen S, McCornack B, et al. Assessing the potential for deep learning and computer vision to identify bumble bee species from images. Scientific Reports 2021 Apr;11(1). http://dx.doi.org/10.1038/s41598-021-87210-1.

[11] Hossain MZ, Ahmed KA, Yoo J. A Machine Learning Based Approach to Study Morphological Features of Bees. In: Proceedings of The 1st International Electronic Conference on Entomology IECE, MDPI; 2021. p. 10607. http://dx.doi.org/10.3390/IECE-10607.

[12] Neyns R, Gardein H, Münzinger M, Hecht R, Greil H, Canters F. Deep learning based mapping of bee-friendly trees through remote sensing: A novel approach to enhance pollinator conservation. Ecological Informatics 2025 Dec;90:103288. http://dx.doi.org/10.1016/j.ecoinf.2025.103288.

[13] Chieffallo L, Torresani M, Zannini P, de Vries JPR, Blaha M, Monacchia A, et al. Machine learning for biodiversity: UAV-based flower detection as an indirect proxy for bee abundance. Ecological Informatics 2025 Nov;91:103346. http://dx.doi.org/10.1016/j.ecoinf.2025.103346.

[14] Mueller TG, Buckner MA. UAV-based Remote Sensing of Bee Nesting Aggregations with Computer Vision for Object Detection. openRxiv 2025 Jun;http://dx.doi.org/10.1101/2025.06.16.659683.

[15] Kwon Y, Lee C, Bak S, Jung C. Proposal of an advanced YOLOX model for real-time detection of Vespa hornets (Hymenoptera; Vespidae), key pests of honey bees. Journal of Asia-Pacific Entomology 2024 Jun;27(2):102234. http://dx.doi.org/10.1016/j.aspen.2024.102234.

[16] Guangnan W, Zhenyou W. A framework for predicting the location of Vespa mandarinia nests via deep learning approach. In: 2022 IEEE Conference on Telecommunications, Optics and Computer Science (TOCS) IEEE; 2022. p. 560–566. http://dx.doi.org/10.1109/TOCS56154.2022.10016104.

[17] Jeon MS, Jeong Y, Lee J, Yu SH, Kim Sb, Kim D, et al. Deep Learning-Based Portable Image Analysis System for Real-Time Detection of Vespa velutina. Applied Sciences 2023 Jun;13(13):7414. http://dx.doi.org/10.3390/app13137414.

[18] María-Luisa PD, Jesús-Ángel RG. Deep Learning for Vespa Velutina Detection. In: 2024 2nd International Conference on Machine Vision, Image Processing Imaging Technology (MVIPIT) IEEE; 2024. p. 216–221. http://dx.doi.org/10.1109/MVIPIT65697.2024.00046.

[19] Singha Roy S, Tingley R, Dorin A. Image background assessment as a novel technique for insect microhabitat identification. Ecological Informatics 2023 Nov;77:102265. http://dx.doi.org/10.1016/j.ecoinf.2023.102265.

[20] Kim B, Pak J, Son HI. Wasp-Hive Candidate Site Search System Using a Small Drone. Entomological Research 2025 Mar;55(3). http://dx.doi.org/10.1111/1748-5967.70034.

[21] Martínez AS, Dreidemie C, Inchaurza F, Cucurull A, Basti M, Masciocchi M. Advancing social insect research through the development of an automated yellowjacket nest activity monitoring station using deep learning. Agricultural and Forest Entomology 2024 Jul;27(1):111–123. http://dx.doi.org/10.1111/afe.12638.

[22] O'Shea-Wheller TA, Corbett A, Osborne JL, Recker M, Kennedy PJ. VespAI: a deep learning-based system for the detection of invasive hornets. Communications Biology 2024 Apr;7(1). http://dx.doi.org/10.1038/s42003-024-05979-z.

[23] Shirali H, Hübner J, Both R, Raupach M, Reischl M, Schmidt S, et al. Image-based recognition of parasitoid wasps using advanced neural networks. Invertebrate Systematics 2024 Jun;38(6). http://dx.doi.org/10.1071/IS24011.

[24] Braga D, Madureira A. Towards a decision support system for the automatic detection of Asian hornets and removal planning. International Journal of Computer Information Systems and Industrial Management Applications 2020;12:8–8.

[25] Chiranjeevi S, Saadati M, Deng ZK, Koushik J, Jubery TZ, Mueller DS, et al. InsectNet: Real-time identification of insects using an end-to-end machine learning pipeline. PNAS Nexus 2024 Dec;4(1). http://dx.doi.org/10.1093/pnasnexus/pgae575.

[26] Rebelo AR, Fagundes JMG, Digiampietri LA, Francoy TM, Bíscaro HH. A fully automatic classification of bee species from wing images. Apidologie 2021 Oct;52(6):1060–1074. http://dx.doi.org/10.1007/s13592-021-00887-1.
